# Supplementary material for: Rapid labelling and covalent inhibition of intracellular native proteins using ligand-directed N-acyl-N-alkyl sulfonamide
Source: Nat Commun. 2018 May 14;9:1870. doi: 10.1038/s41467-018-04343-0 (PMC5951806; doi:10.1038/s41467-018-04343-0)
Supplement: Supplementary file 1 — Supplementary Information [file 41467_2018_4343_MOESM1_ESM.pdf]

Supplementary Information for:

Rapid labelling and covalent inhibition of intracellular native proteins using ligand-directed *N*-acyl-*N*-alkyl sulfonamide

Tamura *et al*.

Supplementary Figures

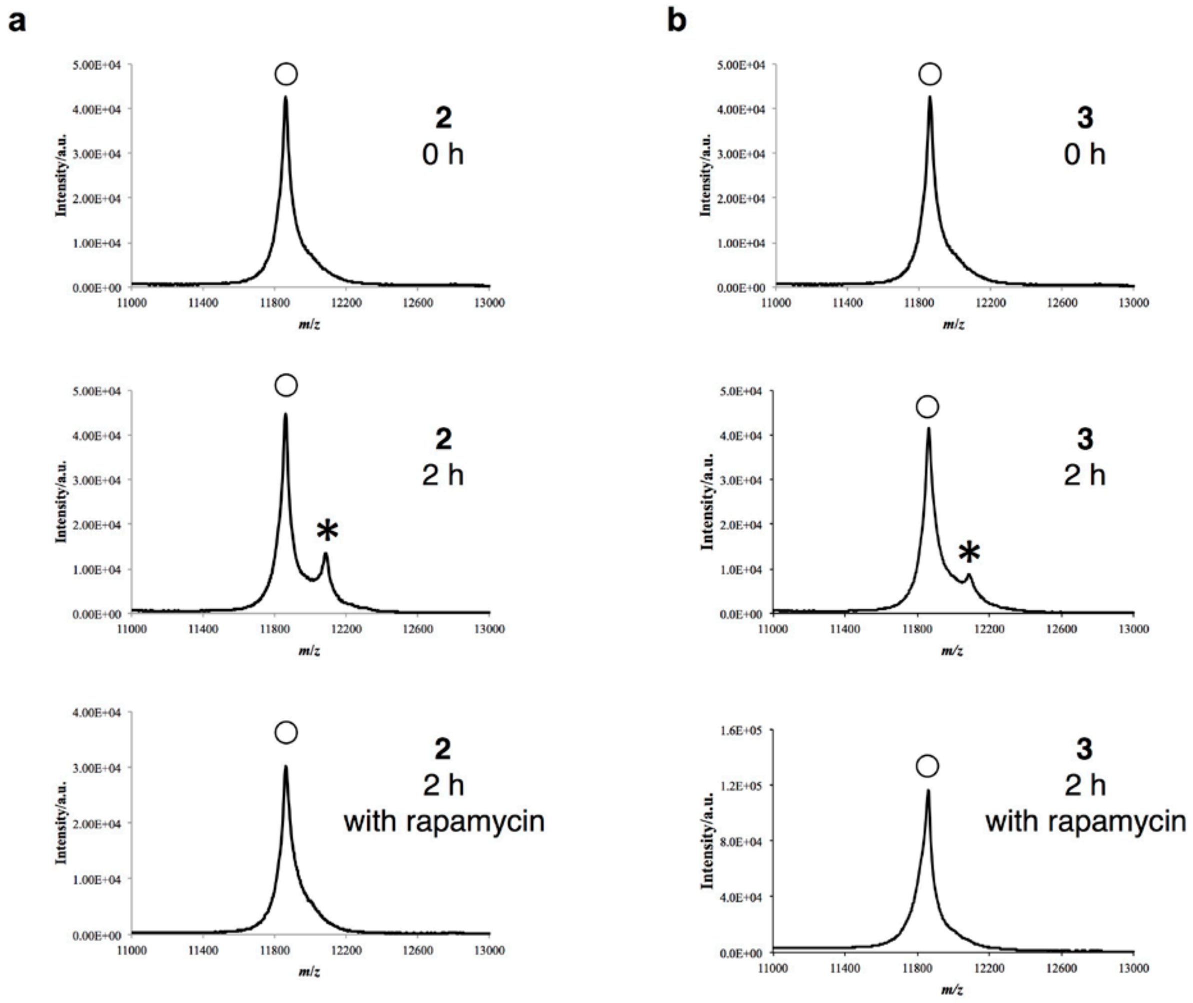

Supplementary Figure 1 *In vitro* labelling of purified FKBP12 with (a) **2** and (b) **3**. Reaction condition: FKBP12 (5 µM), reagent **(**10 µM**)**, rapamycin (50 µM, as an inhibitor), 50 mM HEPES buffer (pH 7.2), 37°C. The reaction was monitored by MALDI-TOF MS. ○, native FKBP12 (M_w : 11 914); *, biotin-labelled FKBP12 (M_w : 12 140).

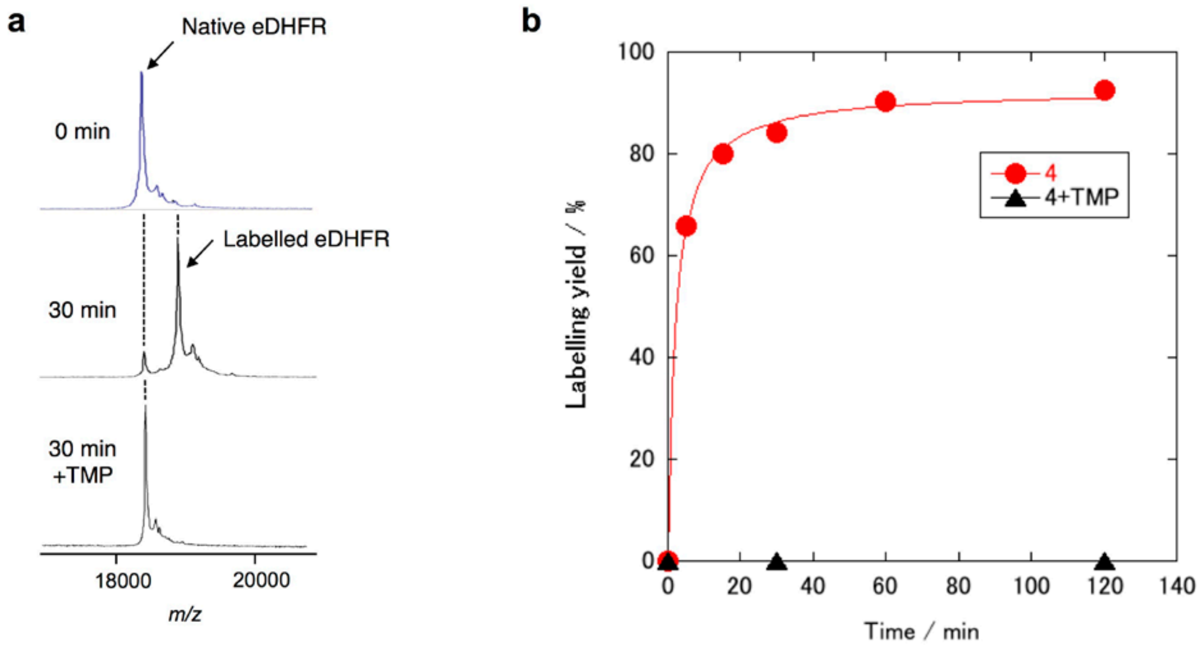

Supplementary Figure 2 *In vitro* labelling of purified eDHFR with reagent **4**. Reaction condition: eDHFR (10 µM), **4 (**20 µM**)**, trimethoprim (TMP) (50 µM, as an inhibitor), 50 mM HEPES buffer (pH 7.2), 37°C. (a) The reaction was monitored by MALDI-TOF MS. Native eDHFR (M_w : 18 512); Labelled eDHFR (M_w : 19 002). (b) Time course of reaction yields of eDHFR labelling by **4** in the absence (●) and in the presence of TMP **(**▲**)**.

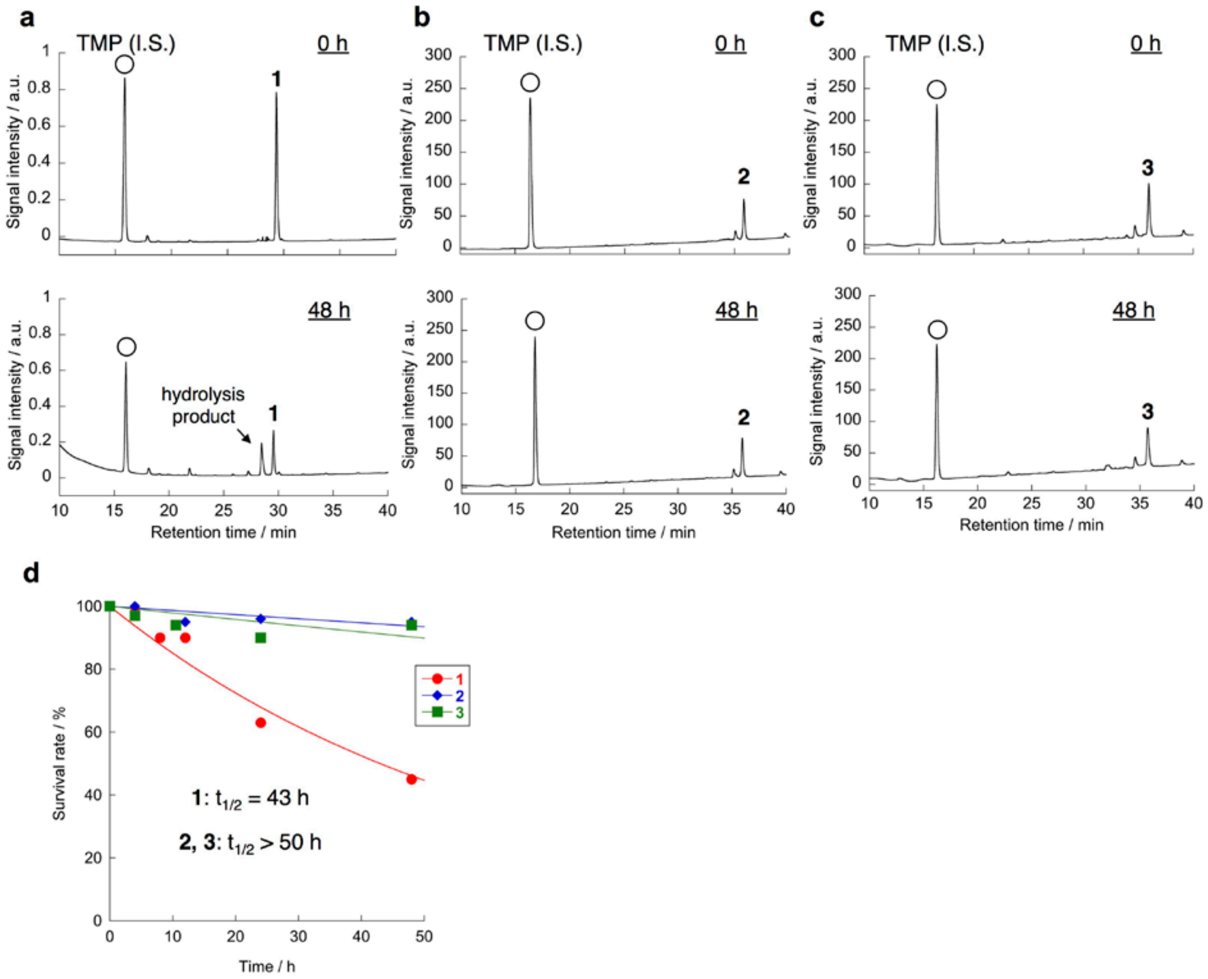

Supplementary Figure 3 HPLC analyses of the stabilities of NASA reagents **1–3** in aqueous buffer. (a–c) HPLC charts of reagent **1** (a), **2** (b) and **3** (c) (each 10 μM) incubated in 50 mM HEPES buffer (pH 7.2) at 37 °C for 0 h (top) and 48 h (bottom). Gradients; A (CH_3CN containing 0.1% TFA) : B (H_2O containing 0.1% TFA) = 5 : 95 (0 min) to 70 : 30 (45 min). ○, Trimethoprim (TMP) as an internal standard (I.S., 16 min). (d) Time course plots of hydrolysis reaction of **1** (●), **2** (◆), and **3** (■). Survival rates at several time points were calculated by the integration of the signal of HPLC analyses (a–c), and fitted by a single-phase exponential decay model to obtain half-life of **1–3** in aqueous buffer.

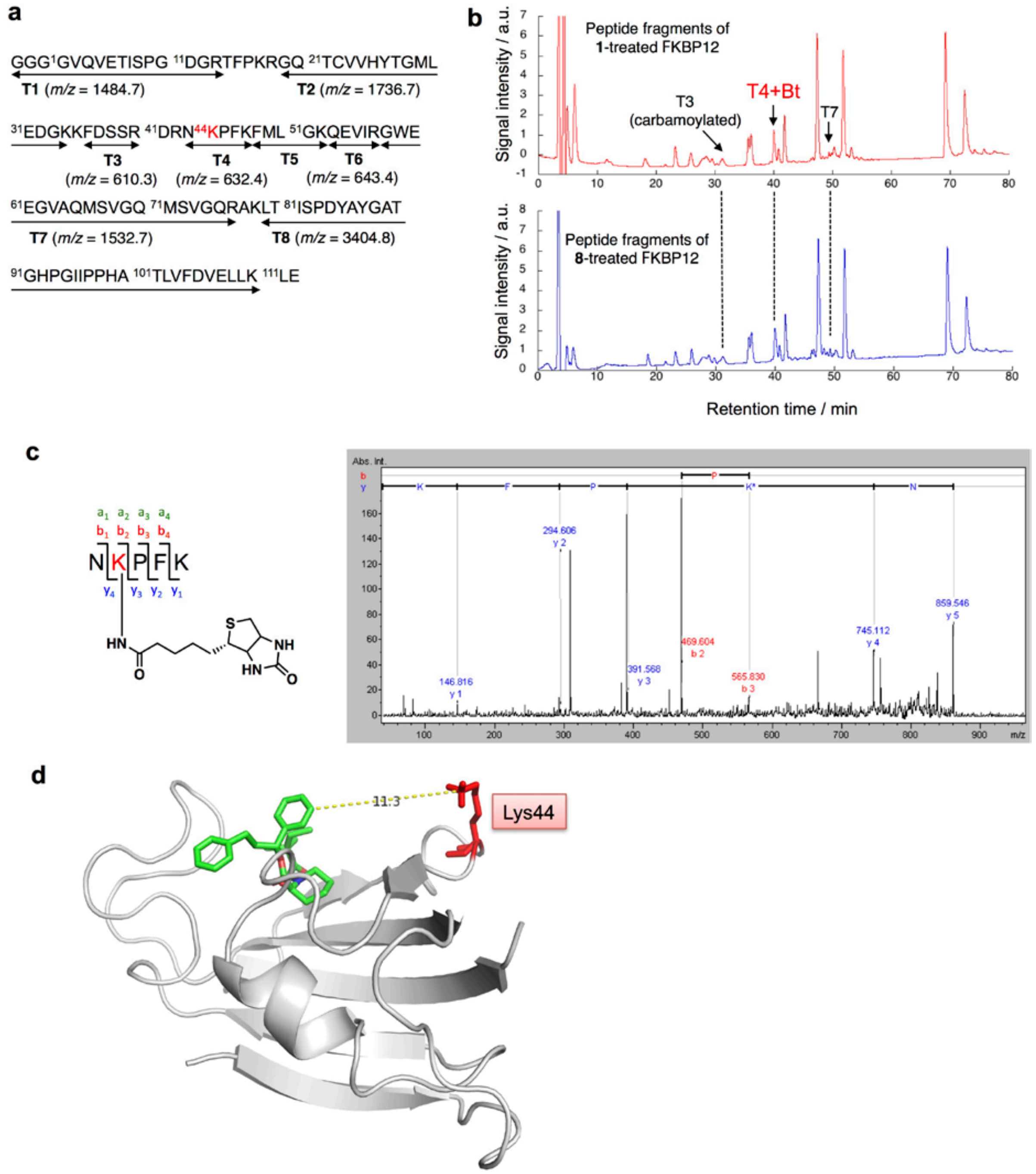

Supplementary Figure 4 Mass spectral analysis of the labelling site of FKBP12 by the reaction with reagent **1** or **8**. (a) The primary sequence of FKBP12 and the predictable fragments generated by tryptic digestion (T1–T8). The Lys 44 labeled with biotin (Bt) is shown in red. (b) HPLC analysis of the digested fragments of (upper) **1**- or (lower) **8**-treated FKBP12. The chromatograms were recorded with UV absorption at 220 nm. Gradients; A (CH_3CN containing 0.1% TFA) : B (H_2O containing 0.1% TFA) = 5 : 95 (0 min) to 55 : 45 (100 min). The peaks marked with the character (e.g. T3) correspond to the peptide fragments, which were characterized by MALDI-TOF MS analysis. MALDI-TOF MS of peak T3

(carbamoylated) (31.2 min): calcd. for $[M+H]^+$ = 654.3, obsd.654.6, peak T4+Bt (40.0 min): calcd. for $[M+H]^+$ = 859.4, obsd. 859.8, peak T7 (49.4 min): calcd. for $[M+H]^+$ = 1532.7, obsd. 1534.4. (d) The crystal structure of FKBP12-ligand (1,3-diphenyl-1-propyl-1-(3,3-dimethyl-1,2-dioxypentyl)-2-piperidine carboxylate) complex (PDB ID: 1FKG[http://dx.doi.org/10.2210/pdb1FKG/pdb]). The labelled residue with **1** and **8** (Lys 44) is highlighted in red, and the ligand is shown in green.

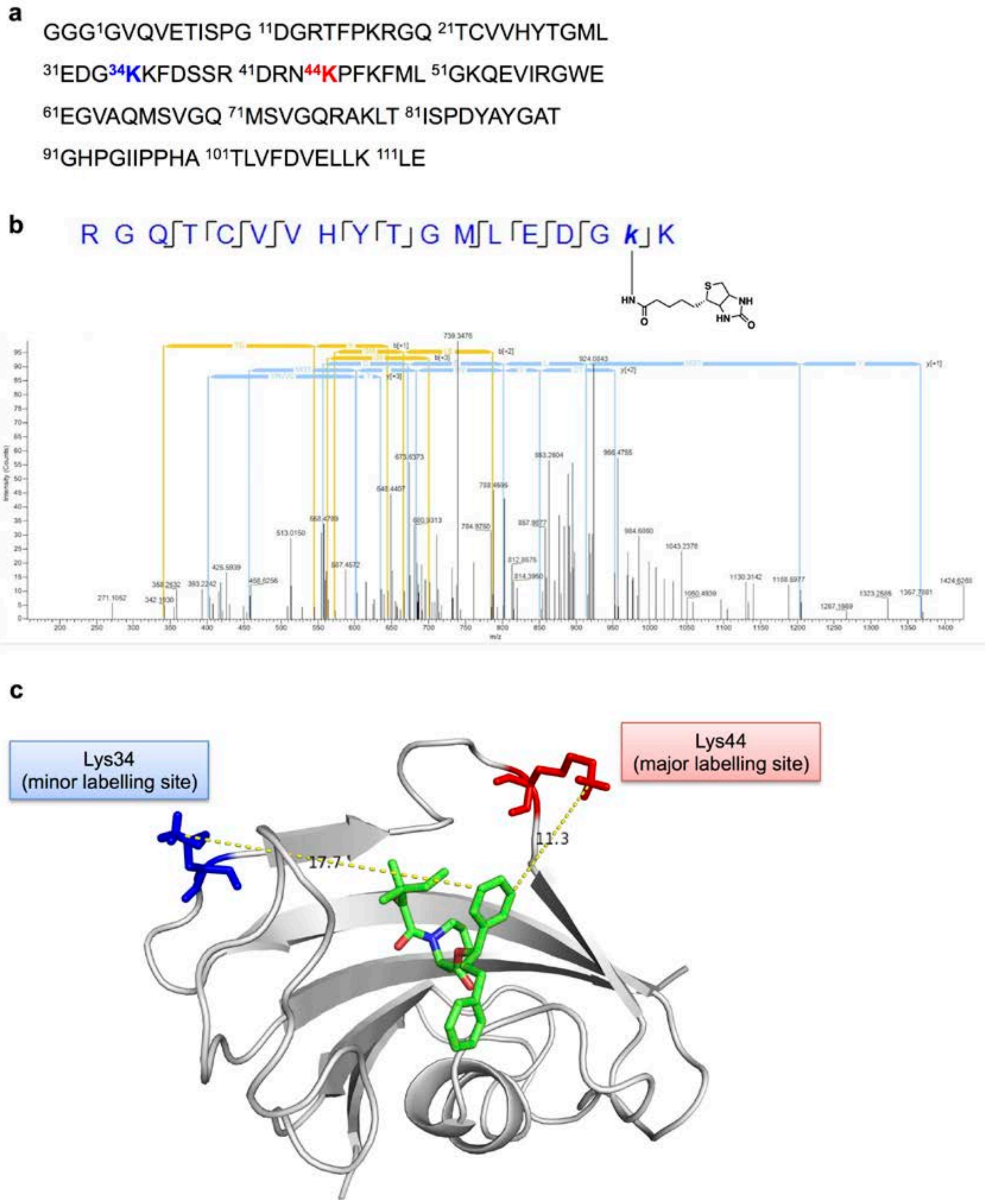

Supplementary Figure 5 Mass spectral analysis of the second labelling site of FKBP12 by the reaction with reagent **1**. Since the second labelling scarcely proceeded, we analyzed the trypsin-digested peptides by more sensitive nanoflow reverse liquid chromatography followed by tandem MS using a LTQ Orbitrap XL hybrid mass spectrometer (Thermo Fisher Scientific). (a) The primary sequence of FKBP12. (b) MSMS spectra of the biotin-modified peptides, RGQTCVVHYTGMLEDG*KK. * indicates the site where biotin-modification was found. Searches allowed three miscleavages by trypsin. (c) The crystal structure of FKBP12 (PDB ID: 1FKG[http://dx.doi.org/10.2210/pdb1FKG/pdb]). Lys44, the major labelling site, is coloured in red. Lys34, the second (minor) labelling site, is coloured in blue. The second labelling site is also located near the ligand-binding pocket (17.7 Å from the bound SLF

ligand), which is consistent with the reaction range of LDNASA reagent **1** (~18 Å length between SLF ligand and the reactive *N*-acyl group). In addition, this labelling was completely abolished in the presence of rapamycin (Figure 2b in the main text). These results clearly indicated that the second labelling also occurred on the basis of the ligand-protein interaction, not random reaction. In the LDNASA chemistry, multiple labelling is possible to proceed because labelling reagents can bind to the ligand-binding pocket of protein after the first labelling by exchange with the cleaved ligand.

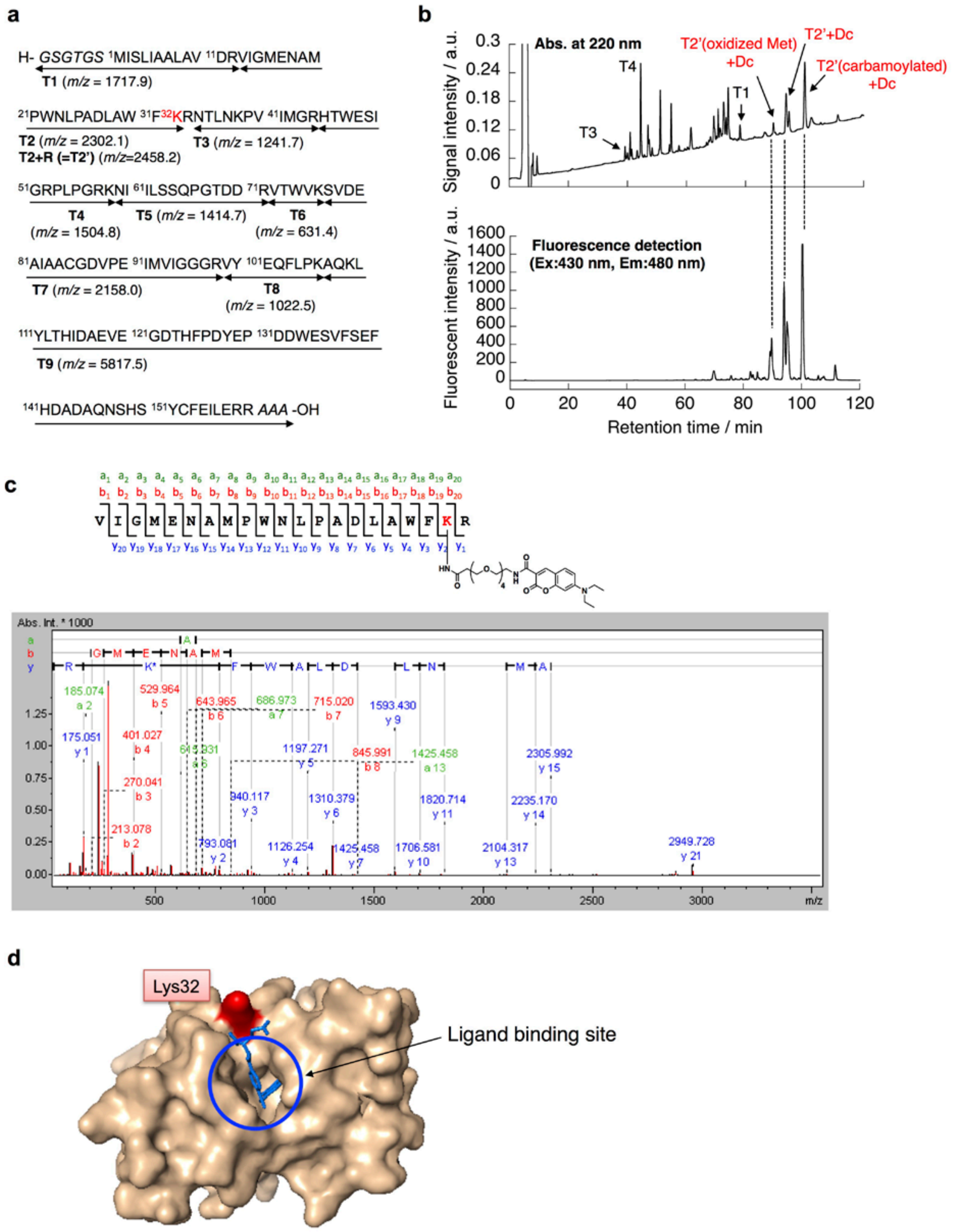

Supplementary Figure 6 Mass spectral analysis of the labelling site of eDHFR by the reaction with reagent **4**. (a) The primary sequence of eDHFR and the predictable fragments generated by tryptic digestion (T1–T9). The Lys 32 labelled with diethylaminocoumarin (Dc) is shown in red. (b) HPLC analysis of the digested fragments of eDHFR. The chromatograms were shown for the fragments derived from the **4**-treated eDHFR detected with UV

absorption at 220 nm (top) and fluorescence (λ_{ex}=430 nm, λ_{em}=480 nm) (bottom). The peaks marked with the character (e.g. T1) correspond to the peptide fragments, which were characterized by MALDI-TOF MS analysis. MALDI-TOF MS of peak T3 (39.8 min): calcd. for $[M+H]^+$ = 1242.7, obsd.1243.1, peak T4 (44.0 min): calcd. for $[M+H]^+$ = 1505.8, obsd.1506.0, peak T1 (78.2 min): calcd. for $[M+H]^+$ = 1718.9, obsd. 1718.7, peak T2' (oxidized Met)+Dc (89.4 min): calcd. for $[M+H]^+$ = 2965.4, obsd. 2966.8, peak T2'+Dc (93.7 min): calcd. for $[M+H]^+$ = 2949.4, obsd. 2949.7, peak T2' (carbamoylated at N-terminus)+Dc (100.0 min): calcd. for $[M+H]^+$ = 2991.4, obsd. 2992.7. (c) MALDI-TOF MS/MS analysis of the T2'+Dc fragment. (d) The crystal structure of eDHFR-MTX (methotrexate) complex (PDB ID: 1RG7[http://dx.doi.org/10.2210/pdb1RG7/pdb]). The labelled residue with **4** (Lys 32) is highlighted in red, and the ligand-binding site is shown in a blue circle.

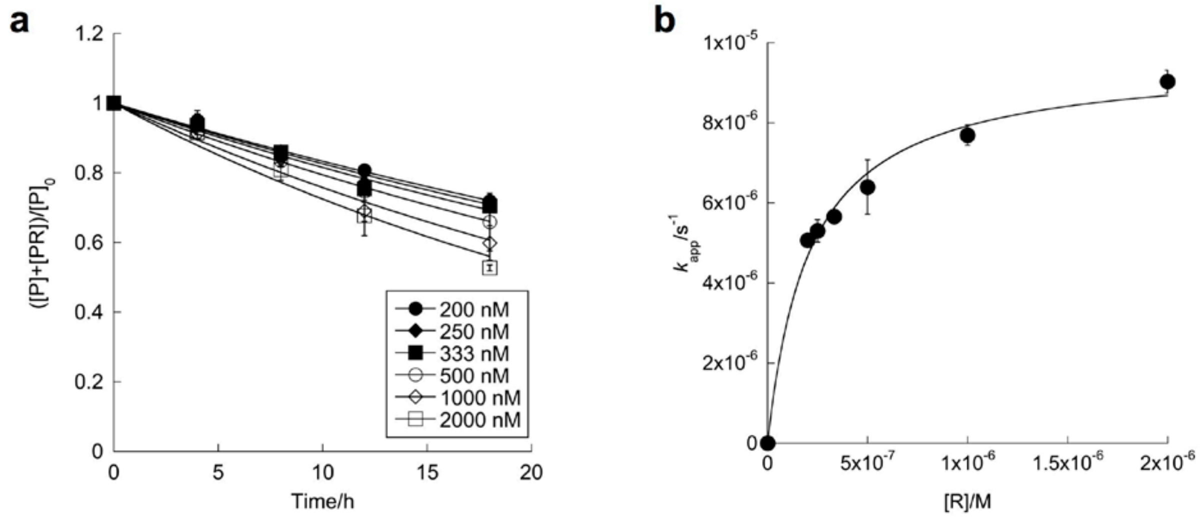

Supplementary Figure 7 Kinetic analysis of FKBP12 labelling with **5** (tosyl reagent). (a) Time courses of the depletion of native (non-labelled) FKBP12 during the labelling reaction with **5**. Purified FKBP12 (100 nM) was incubated with **5** (200–2000 nM) in HEPES buffer (50 mM, pH 7.2) at 37 °C. The reaction was monitored by MALDI-TOF MS. The pseudo-first order reaction rates (k_{app}) were obtained by fitting the data to Supplementary Equation (3). (b) The dependence of k_{app} upon the concentration of **5**. Kinetic parameters were obtained by fitting the data to Supplementary Equation (4). The obtained kinetic parameters were summarized in Table 1 in the main text. Error bars represent standard deviation (s.d.), n = 3.

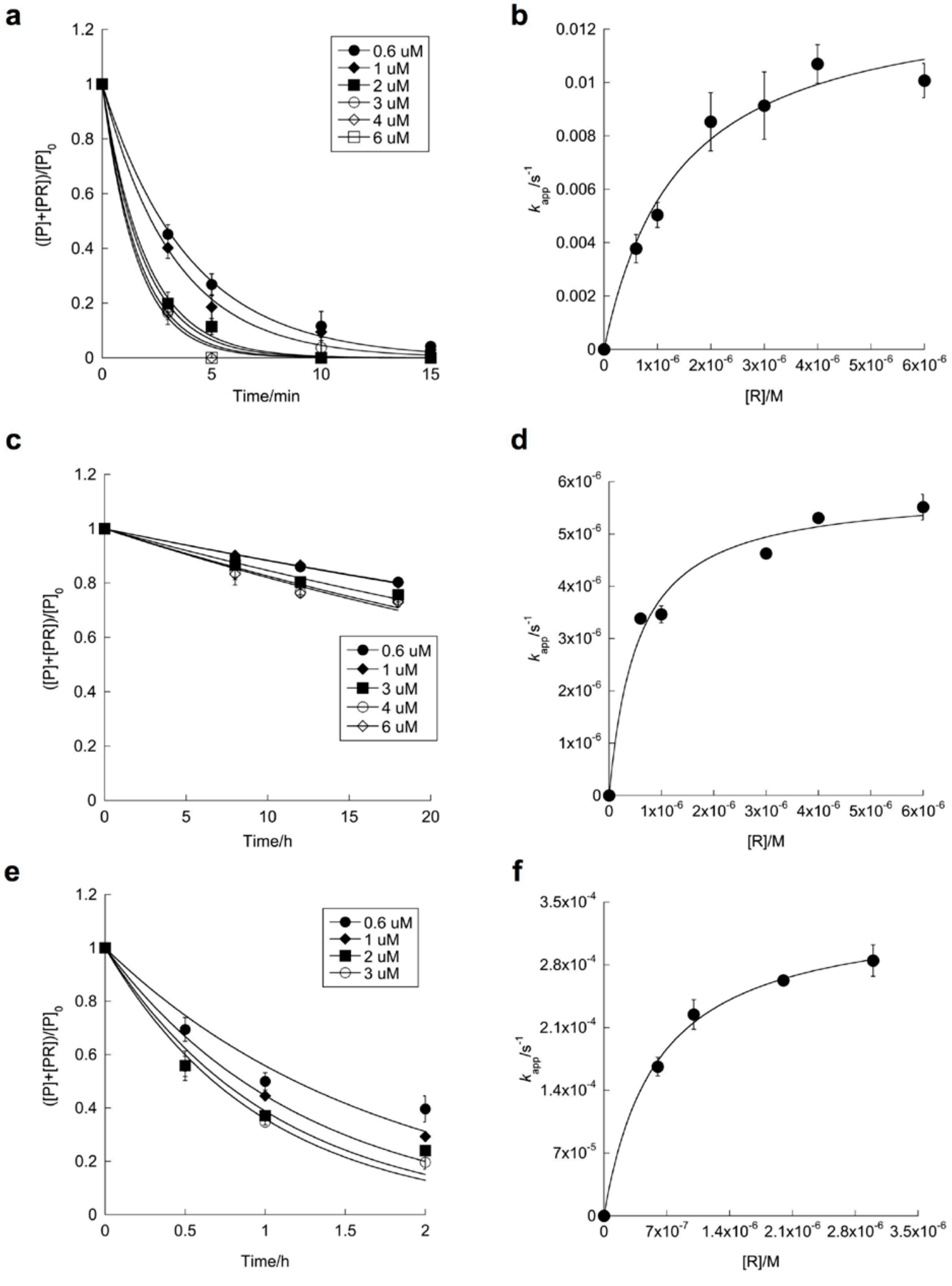

Supplementary Figure 8 Kinetic analysis of eDHFR labelling with **4** (NASA), **6** (alkyloxyacyl imidazole), and **7** (dibromophenylbenzoate). (a, c, e) Time courses of the depletion of native (non-labelled) eDHFR during the labelling reaction with reagent **4** (a), **6** (c), **7** (e). Purified eDHFR (300 nM) was incubated with reagent (0.6–6 μM) in HEPES buffer

(1 mM, pH 7.2) at 37 °C. The pseudo-first order reaction rates (k_{app}) were obtained by fitting the data to Supplementary Equation (3). (b, d, f) The dependence of k_{app} upon the concentration of reagent **4** (b), **6** (d), **7** (f). Kinetic parameters were obtained by fitting the data to Supplementary Equation (4). The obtained kinetic parameters were summarized in Table 1 in the main text. Error bars represent s.d., n = 3.

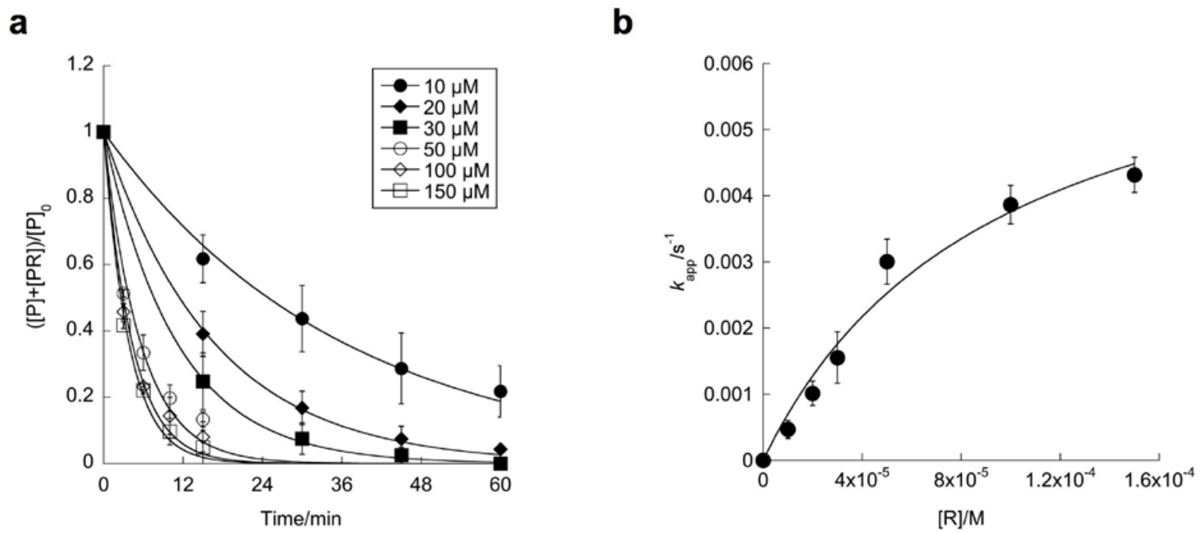

Supplementary Figure 9 Kinetic analysis of FKBP12 labelling with **8** (the low-affinity NASA reagent). (a) Time courses of the depletion of native (non-labelled) FKBP12 during the labelling reaction with **8**. Purified FKBP12 (100 nM) was incubated with **8** (10–150 μM) in HEPES buffer (50 mM, pH 7.2) at 37 °C. The reaction was monitored by MALDI-TOF MS. The pseudo-first order reaction rates (k_{app}) were obtained by fitting the data to Supplementary Equation (3). (b) The dependence of k_{app} upon the concentration of **8**. Kinetic parameters were obtained by fitting the data to Supplementary Equation (4). The obtained kinetic parameters were summarized in Table 1 in the main text. Error bars represent s.d., n = 3.

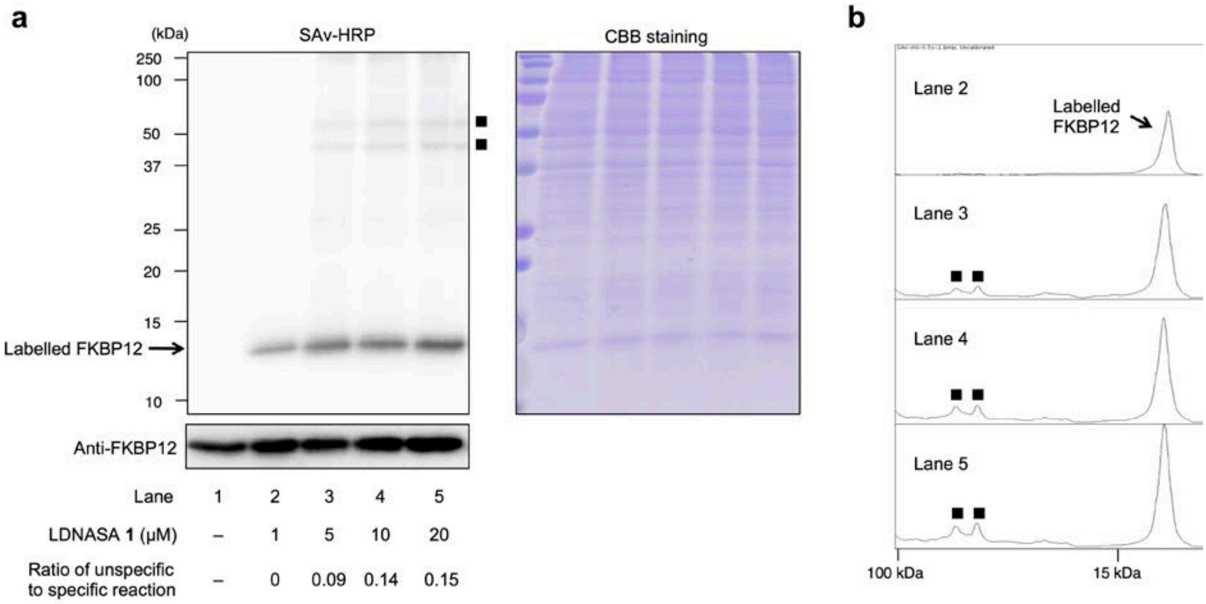

Supplementary Figure 10 Titration experiment of FKBP12 labelling with LDNASA **1** in HeLa cell lysate. (a) Western blotting analysis of labelling reaction. HeLa cell lysate was mixed with recombinant FKBP12 (1 μM) and incubated with reagent (1–20 μM) in 50 mM HEPES buffer, pH 7.2, 37 °C, 1 h. SAv-HRP, streptavidin-horseradish peroxidase conjugate; CBB, Coomassie brilliant blue staining. Unspecific labelling was indicated with a black square. (b) Line plots of signal intensities of lane 2–5 in biotin blotting of Supplementary Figure 10a. The ratios of unspecific to specific reaction were calculated by quantifying areas of labelled FKBP12 and unspecific bands.

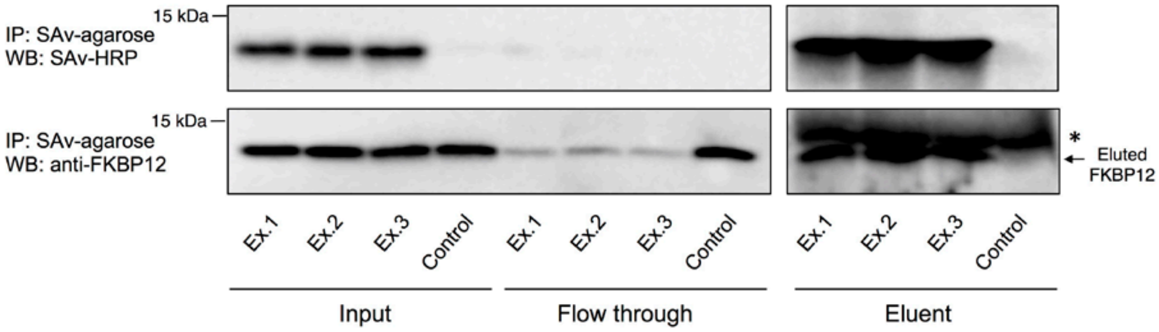

Supplementary Figure 11 Quantification of labelling yields of endogenous FKBP12 in C2C12 cells. After C2C12 cells were incubated with LDNASA **1** (1 µM) for 1 h, the cells were washed twice with PBS and lysed with RIPA buffer (pH 7.4, 25 mM Tris–HCl, 150 mM NaCl, 0.1% SDS, 1% Nonidet P-40, 0.25% deoxycholic acid) containing 1% protease inhibitor cocktail and rapamycin (10 µM), which were aliquoted as input samples. All biotinylated proteins in the lysate were captured with Streptavidin agarose beads (SAv-agarose, Thermo). The flow through and elution fraction, as well as input samples, were subjected to Western blotting analysis using SAv-HRP and anti-FKBP12 antibody. The labelling yields were calculated by the following equation (Supplementary Equation 1):

$$Labelling\ yield\ (\%)\ =\frac{Intensity_{FKBP12,Input} - Intensity_{FKBP12,Flow\ through}}{Intensity_{FKBP12,Input}} \times 100 \quad (1)$$

Experiments were performed in triplicates (Ex.1–Ex.3), and the labelling yields of endogenous FKBP12 in C2C12 cells at 1 h with **1** were estimated to be 78 ± 2 % (mean ± standard deviation). Control, a sample without labelling; *, Nonspecific band derived from Streptavidin monomer.

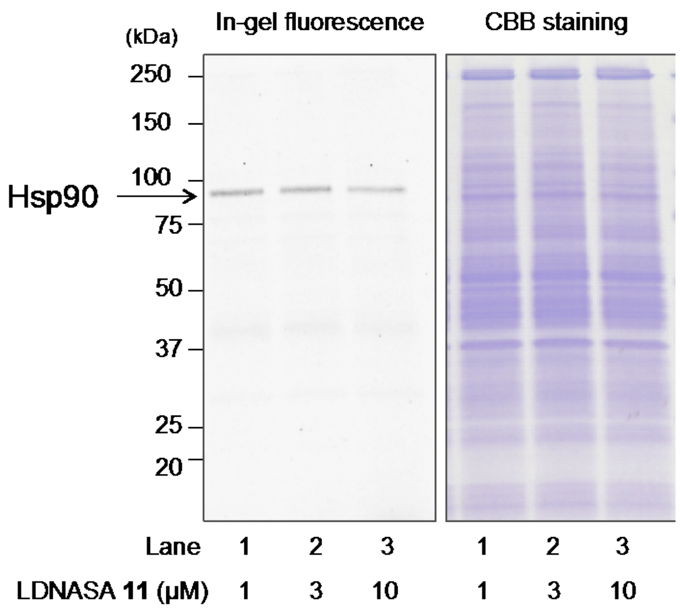

Supplementary Figure 12 SDS-PAGE analysis of endogenous Hsp90 labelling in live SKBR3 cells with high concentrations of LDNASA **11**. The cells were treated with **11** (1–10 μM) for 3 h at 37 °C in medium (pH 7.4). After washing, the cells were lysed and analyzed by in-gel fluorescence and Coomassie brilliant blue (CBB) staining. No increase in unspecific reaction was detected in this concentration range.

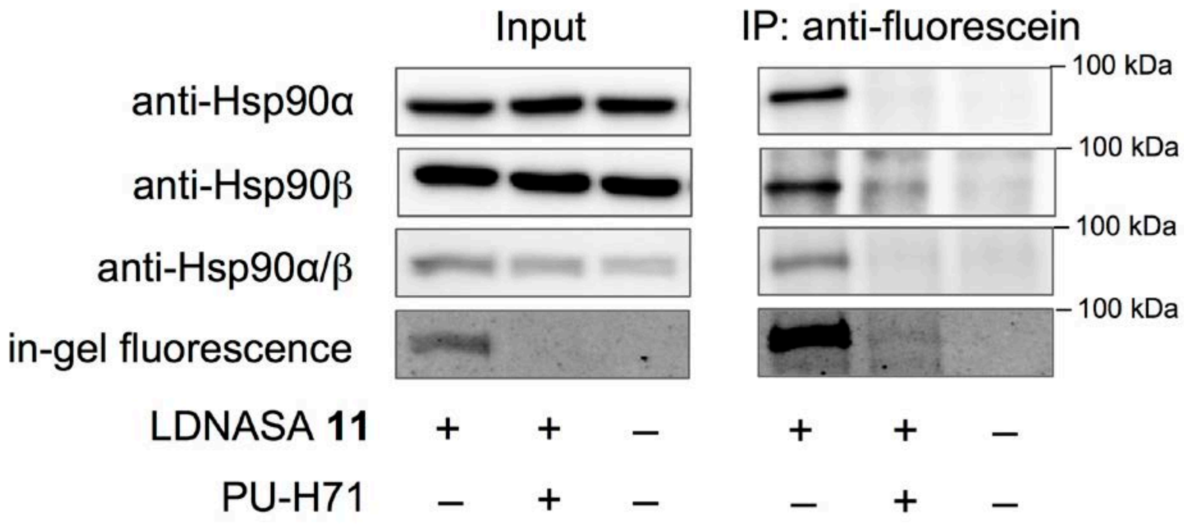

Supplementary Figure 13 Isoform selectivity of Hsp90 labelling with LDNASA **11** in SKBR3 cells. The cells (1.0×10^6 cells) were treated with LDNASA **11** (1 µM) in the absence or presence of PU-H71 (10 µM) for 3 h at 37 °C in serum-free medium. After lysis of the cells, the lysate was immunoprecipitated with Dynabeads Protein G (Thermo Fisher Scientific) using anti-fluorescein antibody (Abcam, ab19491). The immunoprecipitates were analyzed by in-gel fluorescence scanning and western blotting using anti-Hsp90α (Cell Signaling Technology (CST), #8165), anti-Hsp90β (CST, #7411) and anti-Hsp90 (both α and β)-HRP conjugate (CST, #79641).

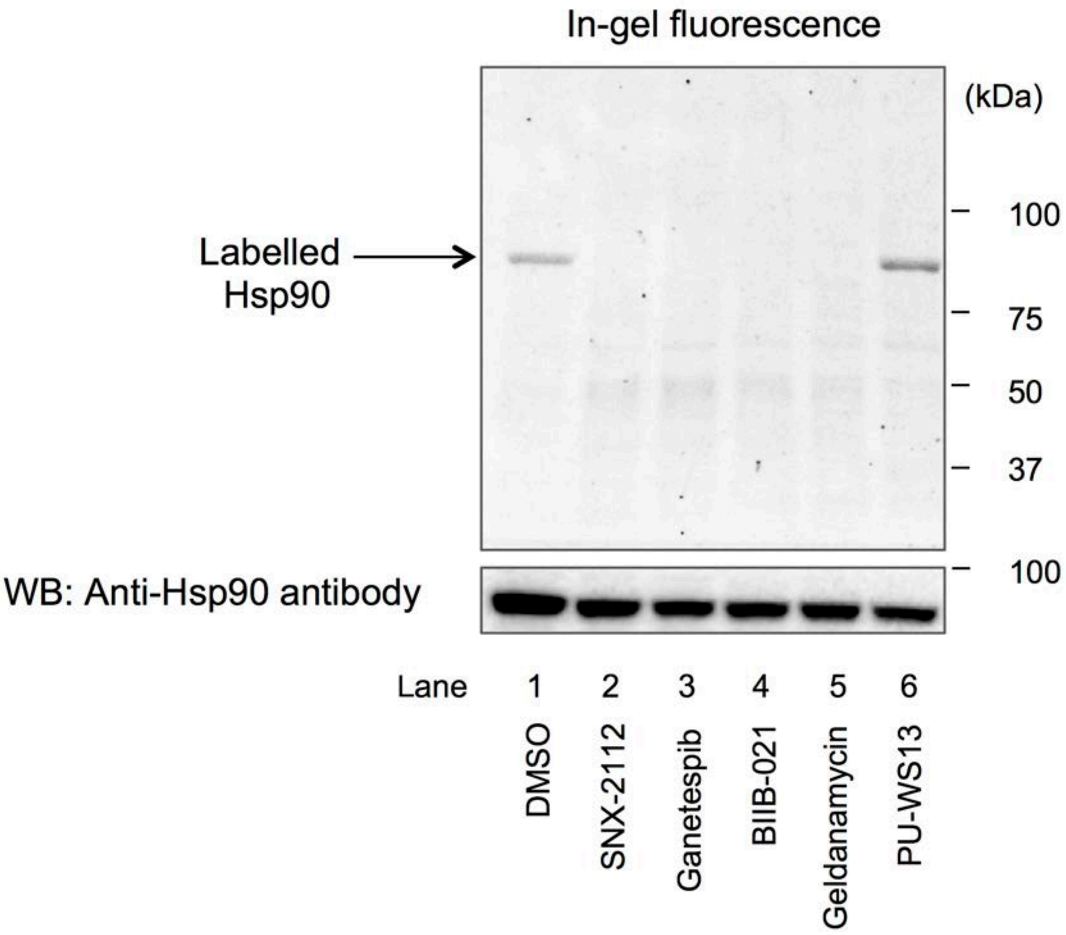

Supplementary Figure 14 SDS-PAGE analysis of endogenous Hsp90 labelling in live HeLa cells with LDNASA **11** (0.5 µM) in the presence of 10 µM of competitive Hsp90 inhibitors. Lane 1, DMSO control; lane 2, SNX-2112 (IC_{50} = 29 nM); lane 3, Ganetespib (IC_{50} = 5 nM); lane 4, BIIB-021 (IC_{50} = 19 nM); lane5, Geldanamycin (IC_{50} = 28 nM); lane 6, PU-WS13 (IC_{50} = 27 µM). IC_{50} values described here are reported for Hsp90α in Supplementary References 1 and 2. PU-WS13 showed less potent inhibition of the reaction presumably due to its low affinity for Hsp90.

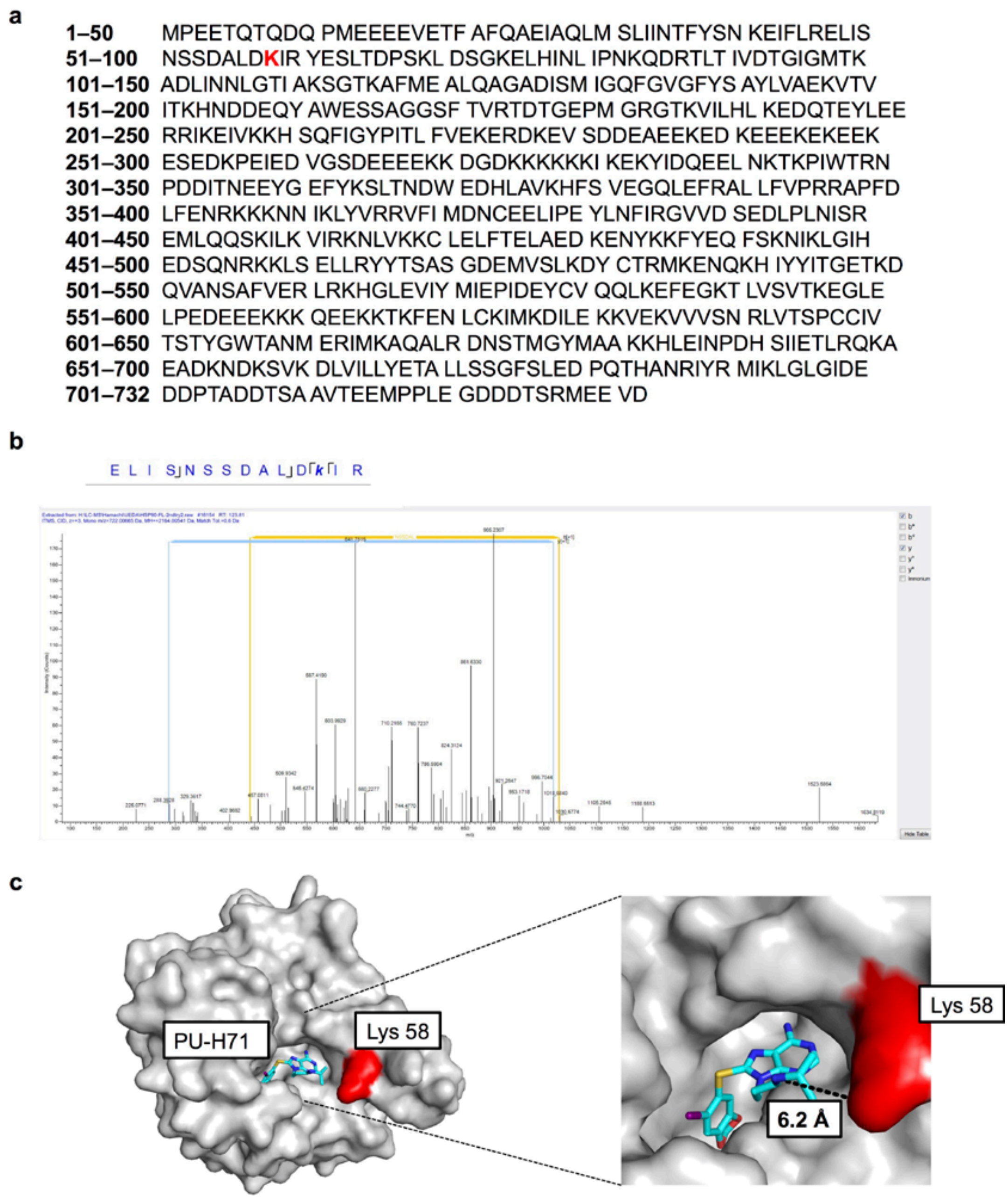

Supplementary Figure 15 LC-MSMS analysis of fluorescein-modified peptides digested from endogenous Hsp90 labelled with LDNASA **11**. (a) The primary sequence of Hsp90α. The Lys 58 labelled with fluorescein is shown in red. (b) MSMS spectra of the fluorescein-modified peptides, ELISNSSDALD*KIR. * indicates the site where fluorescein-modification was found. (c) The crystal structure of N-terminal domain of Hsp90α–PU-H71 complex (PDB ID: 2FWZ[http://dx.doi.org/10.2210/pdb2FWZ/pdb]). The modified residue with **11** and **12** (Lys 58) is highlighted in red, and the PU-H71 ligand is colored in light blue.

```
P07900 HS90A_HUMAN  1    MPEETQTQDQPMEEEEVETFAFQAEIAQLMSLIINTFYSNKEIFLRELISNSSDALDKIR  60
                         MPEE         EEEVETFAFQAEIAQLMSLIINTFYSNKEIFLRELISN+SDALDKIR
P08238 HS90B_HUMAN  1    MPEEVHHG-----EEEVETFAFQAEIAQLMSLIINTFYSNKEIFLRELISNASDALDKIR  55

P07900 HS90A_HUMAN  61   YESLTDPSKLDSGKELHINLIPNKQDRTLTIVDTGIGMTKADLINNLGTIAKSGTKAFME  120
                         YESLTDPSKLDSGKEL I++IPN Q+RTLT+VDTGIGMTKADLINNLGTIAKSGTKAFME
P08238 HS90B_HUMAN  56   YESLTDPSKLDSGKELKIDIIPNPQERTLTLVDTGIGMTKADLINNLGTIAKSGTKAFME  115

P07900 HS90A_HUMAN  121  ALQAGADISMIGQFGVGFYSAYLVAEKVTVITKHNDDEQYAWESSAGGSFTVRTDTGEPM  180
                         ALQAGADISMIGQFGVGFYSAYLVAEKV VITKHNDDEQYAWESSAGGSFTVR D GEP+
P08238 HS90B_HUMAN  116  ALQAGADISMIGQFGVGFYSAYLVAEKVVVITKHNDDEQYAWESSAGGSFTVRADHGEPI  175

P07900 HS90A_HUMAN  181  GRGTKVILHLKEDQTEYLEERRIKEIVKKHSQFIGYPITLFVEKERDKEVSDDEAEEKED  240
                         GRGTKVILHLKEDQTEYLEERR+KE+VKKHSQFIGYPITL++EKER+KE+SDDEAEE++
P08238 HS90B_HUMAN  176  GRGTKVILHLKEDQTEYLEERRVKEVVKKHSQFIGYPITLYLEKEREKEISDDEAEEEKG  235

P07900 HS90A_HUMAN  241  KEEEKEKEEKESEDKPEIEDVGSDEEEEKKDGDKKKKKKIKEKYIDQEELNKTKPIWTRN  300
                            EKE+E+K+ E+KP+IEDVGSDEE++     KKK KKIKEKYIDQEELNKTKPIWTRN
P08238 HS90B_HUMAN  236  ---EKEEEDKDDEEKPKIEDVGSDEEDDSGKDKKKKTKKIKEKYIDQEELNKTKPIWTRN  292

P07900 HS90A_HUMAN  301  PDDITNEEYGEFYKSLTNDWEDHLAVKHFSVEGQLEFRALLFVPRRAPFDLFENRKKKNN  360
                         PDDIT EEYGEFYKSLTNDWEDHLAVKHFSVEGQLEFRALLF+PRRAPFDLFEN+KKKNN
P08238 HS90B_HUMAN  293  PDDITQEEYGEFYKSLTNDWEDHLAVKHFSVEGQLEFRALLFIPRRAPFDLFENKKKKNN  352

P07900 HS90A_HUMAN  361  IKLYVRRVFIMDNCEELIPEYLNFIRGVVDSEDLPLNISREMLQQSKILKVIRKNLVKKC  420
                         IKLYVRRVFIMD+C+ELIPEYLNFIRGVVDSEDLPLNISREMLQQSKILKVIRKN+VKKC
P08238 HS90B_HUMAN  353  IKLYVRRVFIMDSCDELIPEYLNFIRGVVDSEDLPLNISREMLQQSKILKVIRKNIVKKC  412

P07900 HS90A_HUMAN  421  LELFTELAEDKENYKKFYEQFSKNIKLGIHEDSQNRKKLSELLRYYTSASGDEMVSLKDY  480
                         LELF+ELAEDKENYKKFYE FSKN+KLGIHEDS NR++LSELLRY+TS SGDEM SL +Y
P08238 HS90B_HUMAN  413  LELFSELAEDKENYKKFYEAFSKNLKLGIHEDSTNRRRLSELLRYHTSQSGDEMTSLSEY  472

P07900 HS90A_HUMAN  481  CTRMKENQKHIYYITGETKDQVANSAFVERLRKHGLEVIYMIEPIDEYCVQQLKEFEGKT  540
                          +RMKE QK IYYITGE+K+QVANSAFVER+RK G EV+YM EPIDEYCVQQLKEF+GK+
P08238 HS90B_HUMAN  473  VSRMKETQKSIYYITGESKEQVANSAFVERVRKRGFEVVYMTEPIDEYCVQQLKEFDGKS  532

P07900 HS90A_HUMAN  541  LVSVTKEGLELPEDEEEKKKQEEKKTKFENLCKIMKDILEKKVEKVVVSNRLVTSPCCIV  600
                         LVSVTKEGLELPEDEEEKKK EE K KFENLCK+MK+IL+KKVEKV +SNRLV+SPCCIV
P08238 HS90B_HUMAN  533  LVSVTKEGLELPEDEEEKKKMEESKAKFENLCKLMKEILDKKVEKVTISNRLVSSPCCIV  592

P07900 HS90A_HUMAN  601  TSTYGWTANMERIMKAQALRDNSTMGYMAAKKHLEINPDHSIIETLRQKAEADKNDKSVK  660
                         TSTYGWTANMERIMKAQALRDNSTMGYM AKKHLEINPDH I+ETLRQKAEADKNDK+VK
P08238 HS90B_HUMAN  593  TSTYGWTANMERIMKAQALRDNSTMGYMMAKKHLEINPDHPIVETLRQKAEADKNDKAVK  652

P07900 HS90A_HUMAN  661  DLVILLYETALLSSGFSLEDPQTHANRIYRMIKLGLGIDEDDPTADDTSAAVTEEMPPLE  720
                         DLV+LL+ETALLSSGFSLEDPQTH+NRIYRMIKLGLGIDED+  A++ +AAV +E+PPLE
P08238 HS90B_HUMAN  653  DLVVLLFETALLSSGFSLEDPQTHSNRIYRMIKLGLGIDEDEVAAEEPNAAVPDEIPPLE  712

P07900 HS90A_HUMAN  721  GDDDTSRMEEVD                                                  732
                         GD+D SRMEEVD
P08238 HS90B_HUMAN  713  GDEDASRMEEVD                                                  724
```

Supplementary Figure 16 Sequence homology of human Hsp90α (Uniprot accession No. P07900) and Hsp90β (Uniprot accession No. P08238). Sequence alignment was performed by BLAST. The labelled Lys58 of Hsp90α and the corresponding Lys53 of Hsp90β are enclosed in a red square.

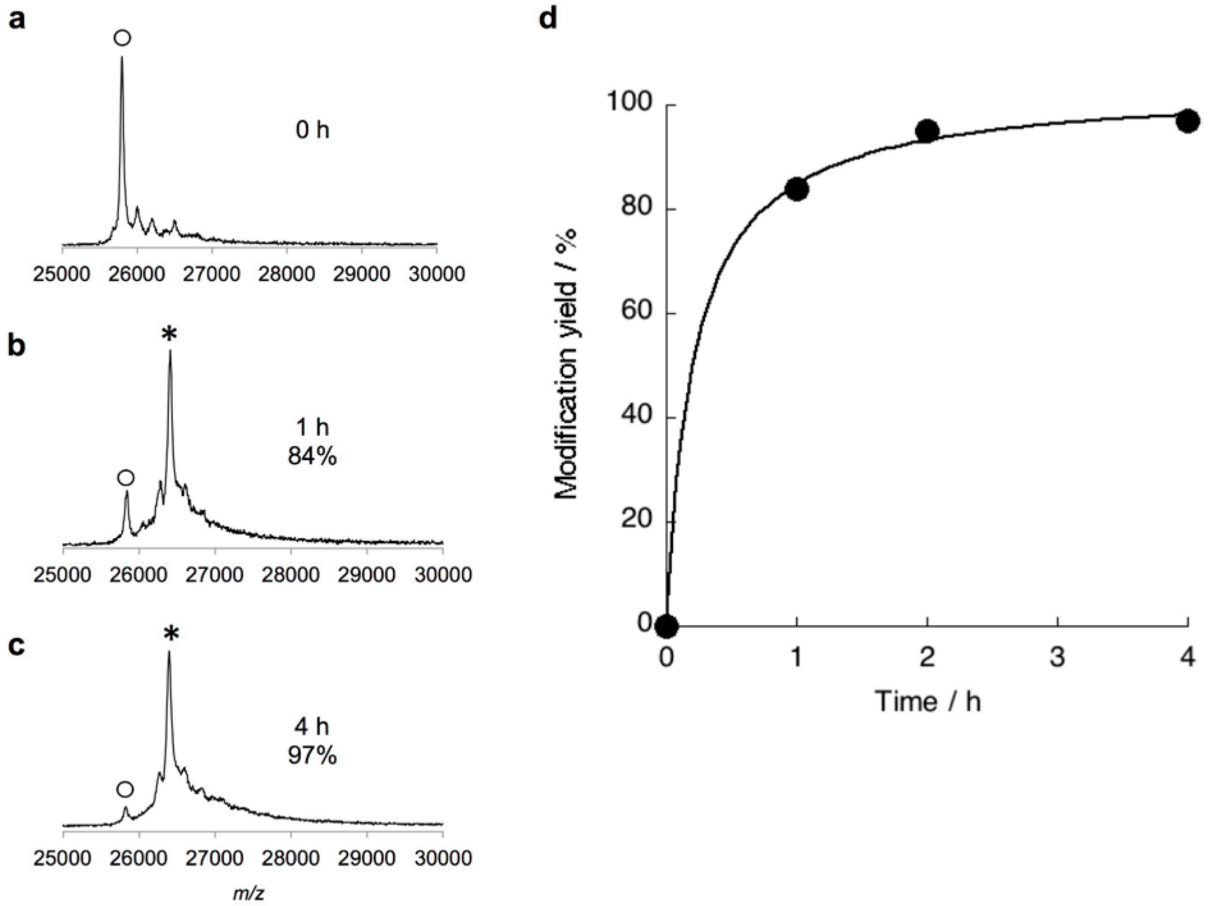

Supplementary Figure 17 MALDI-TOF MS analysis of recombinant N-terminal ATP binding domain of Hsp90α treated with compound **12**. Reaction conditions: 6.4 µM N-terminal domain of Hsp90α, 10 µM compound **12**, 50 mM HEPES buffer, pH 7.2, 37 °C, 0 h (a), 1 h (b), and 4 h (c). ○, native N-terminal domain of Hsp90 (M_w : 25 832); *, covalent adduct of the N-terminal domain with **12** (M_w : 26 400). (d) Time profile of the covalent bond formation with **12**.

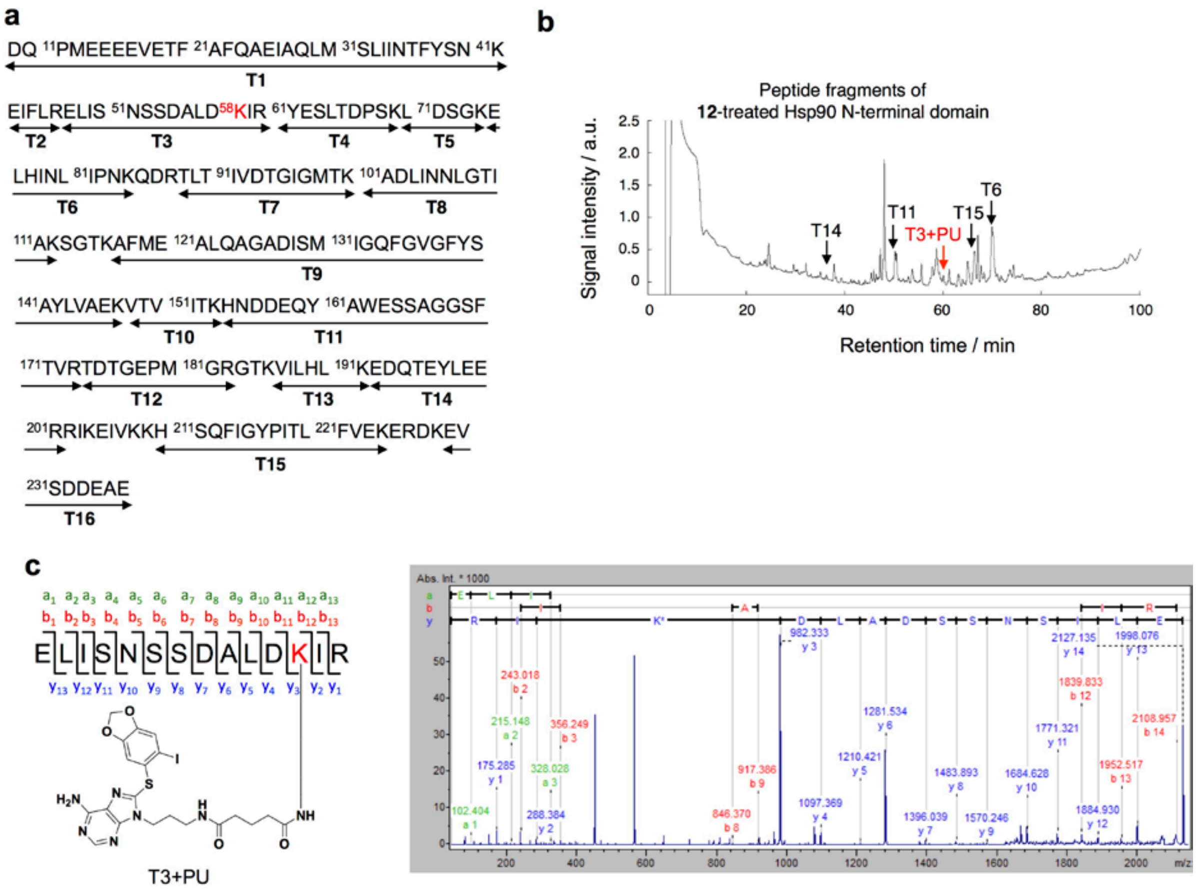

Supplementary Figure 18 Mass spectral analysis of the modification site of N-terminal ATP binding domain of Hsp90α by the reaction with compound **12**. (a) The primary sequence of N-terminal domain of Hsp90α and the predictable fragments generated by tryptic digestion (T1–T16). The Lys 58 modified with **12** is shown in red. (b) HPLC analysis of the digested fragments of the N-terminal domain. The chromatogram was shown for the fragments derived from the **12**-treated N-terminal domain detected with UV absorption at 220 nm. The peaks marked with the character (e.g. T6) correspond to the peptide fragments, which were characterized by MALDI-TOF MS analysis. MALDI-TOF MS of peak T14 (36.2 min): calcd. for $[M+H]^+$ = 1311.6, obsd.1311.6, peak T11 (49.8 min): calcd. for $[M+H]^+$ = 2256.0, obsd. 2255.9, peak T3+PU (modified with **12**) (60.0 min): calcd. for $[M+H]^+$ = 2126.8, obsd. 2126.8, peak T15 (66.5 min): calcd. for $[M+H]^+$ = 1778.9, obsd. 1778.9, peak T6 (71.7 min): calcd. for $[M+H]^+$ = 1191.0, obsd. 1192.0. (c) MALDI-TOF MS/MS analysis of the peptide fragment modified with **12**.

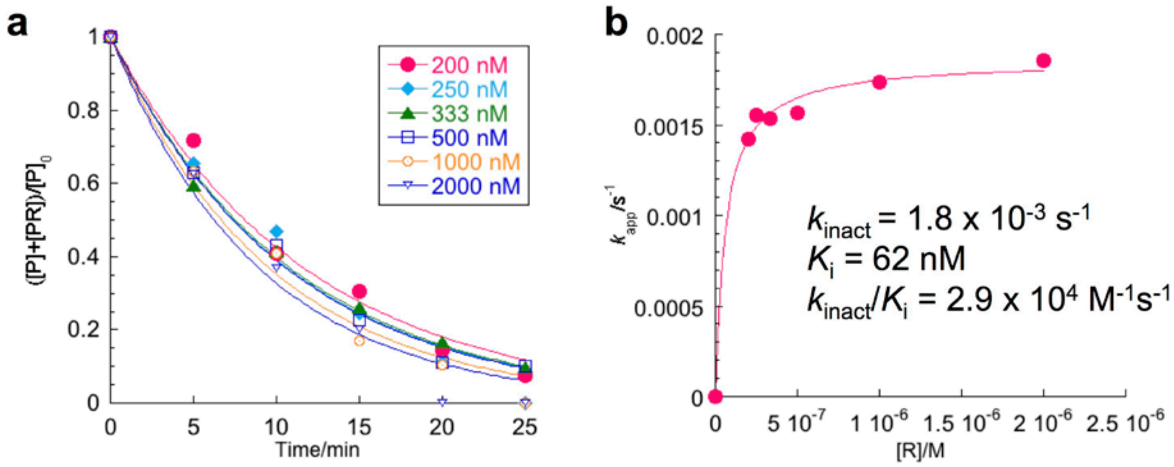

Supplementary Figure 19 Kinetic analysis of covalent inhibition of Hsp90 with **12**. (a) Time courses of the depletion of native (non-labelled) N-terminal ATP binding domain of Hsp90α during the labelling reaction with compound **12**. Purified N-terminal ATP binding domain of Hsp90α (100 nM) was incubated with **12** (200–2000 nM) in PBS buffer (pH 7.2) at 37 °C. The pseudo-first order reaction rates (k_{app}) were obtained by fitting the data to Supplementary Equation (3). (b) The dependence of k_{app} upon the concentration of reagent **12**. Kinetic parameters for covalent inhibition (k_{inact} and K_i) were obtained by fitting the data to Supplementary Equation (4).

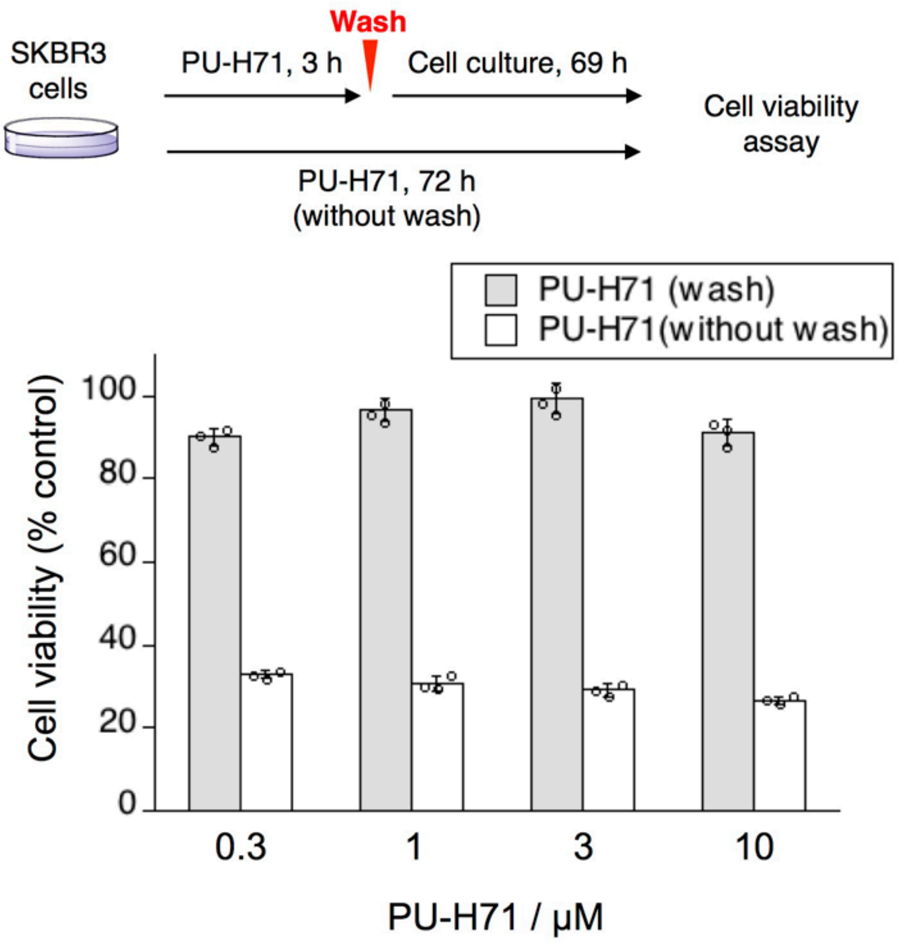

Supplementary Figure 20 Viability of the cells 69 h after PU-H71 washout (grey bars), or 72 h-treatment with PU-H71 (black bars). Error bars represent s.d., n = 3.

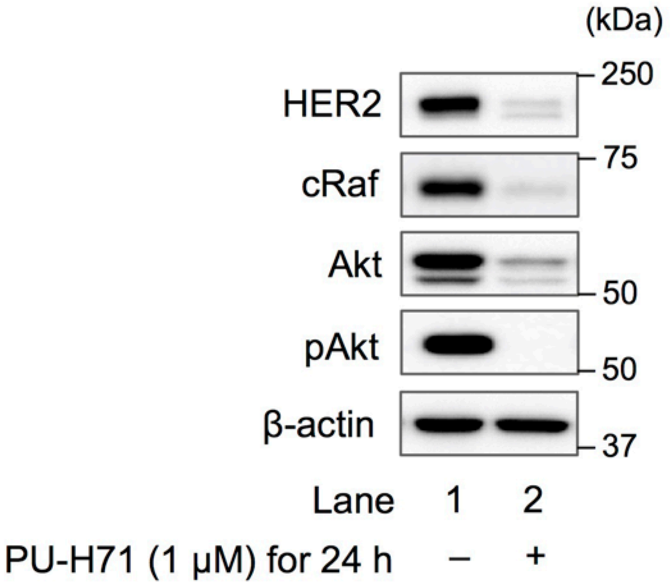

Supplementary Figure 21 Western blotting analysis of the destabilization of cellular client proteins induced by treatment of PU-H71 (1 μM) for 24 h (without washing). β-actin is a control as a non-Hsp90 client protein.

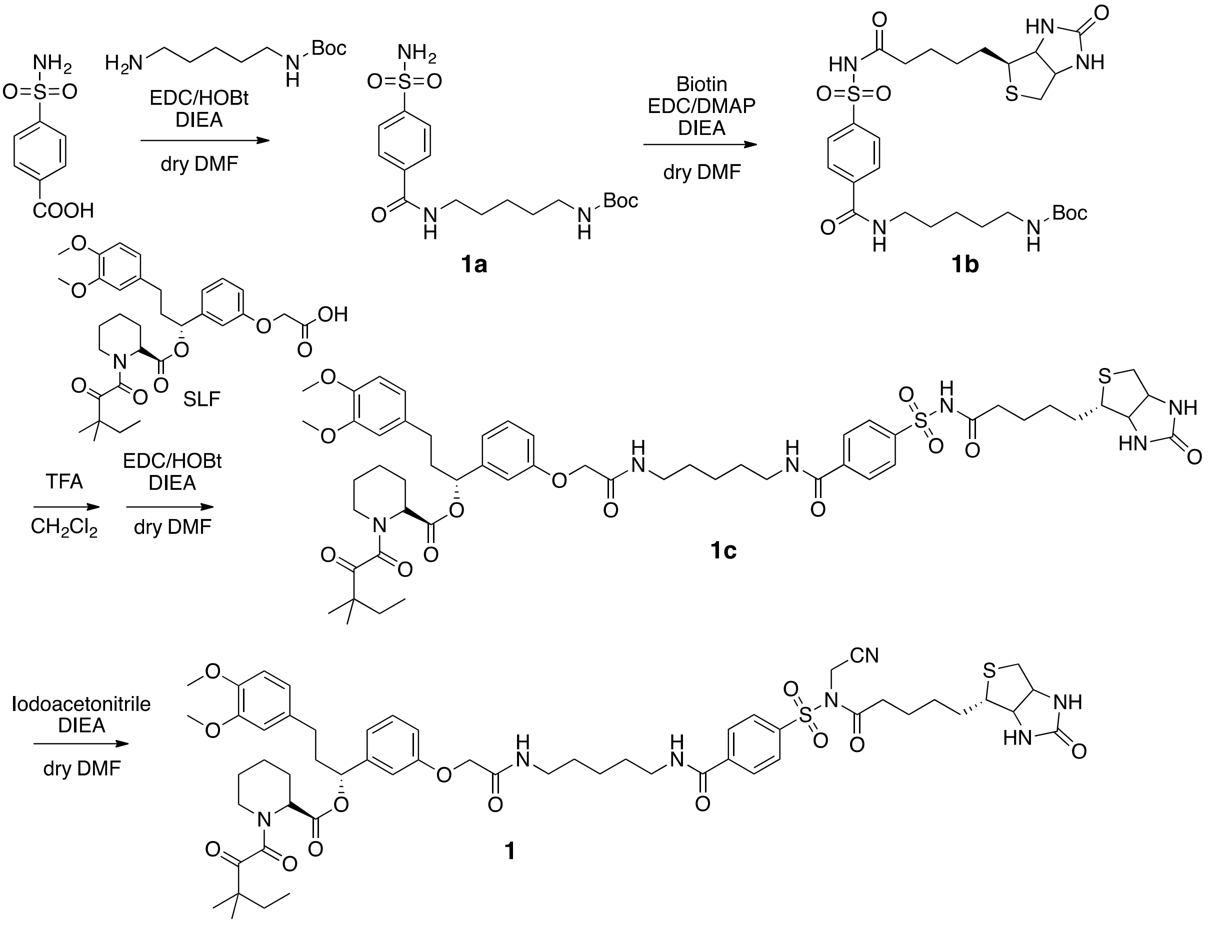

Supplementary Figure 22 Synthetic scheme of compound **1**

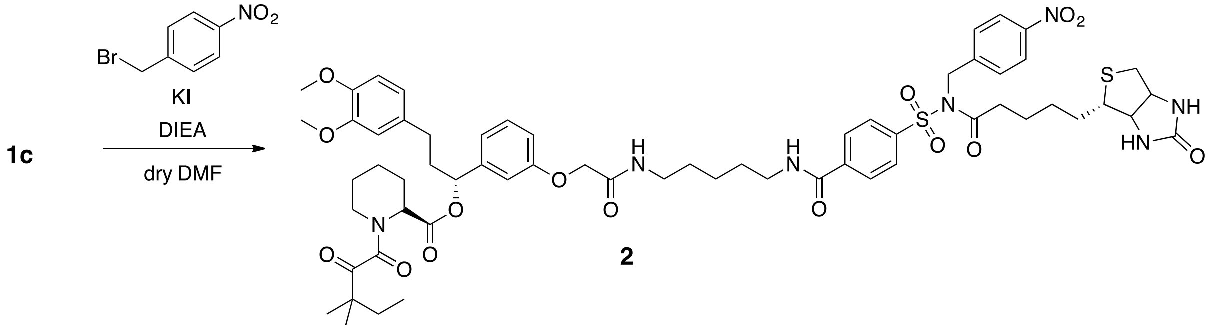

Supplementary Figure 23 Synthetic scheme of compound **2**

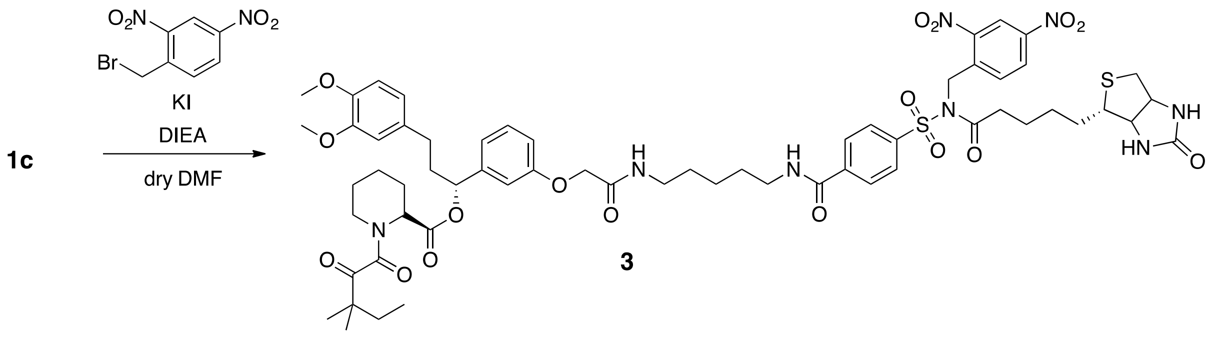

Supplementary Figure 24 Synthetic scheme of compound **3**

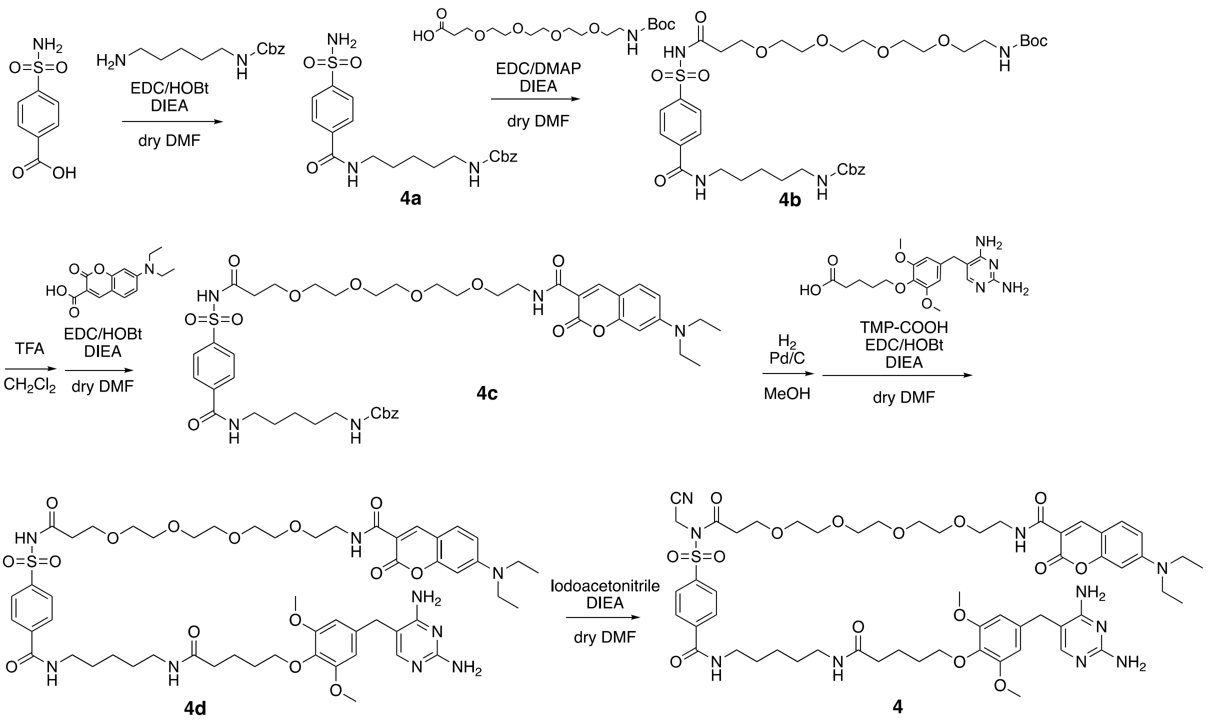

Supplementary Figure 25 Synthetic scheme of compound **4**

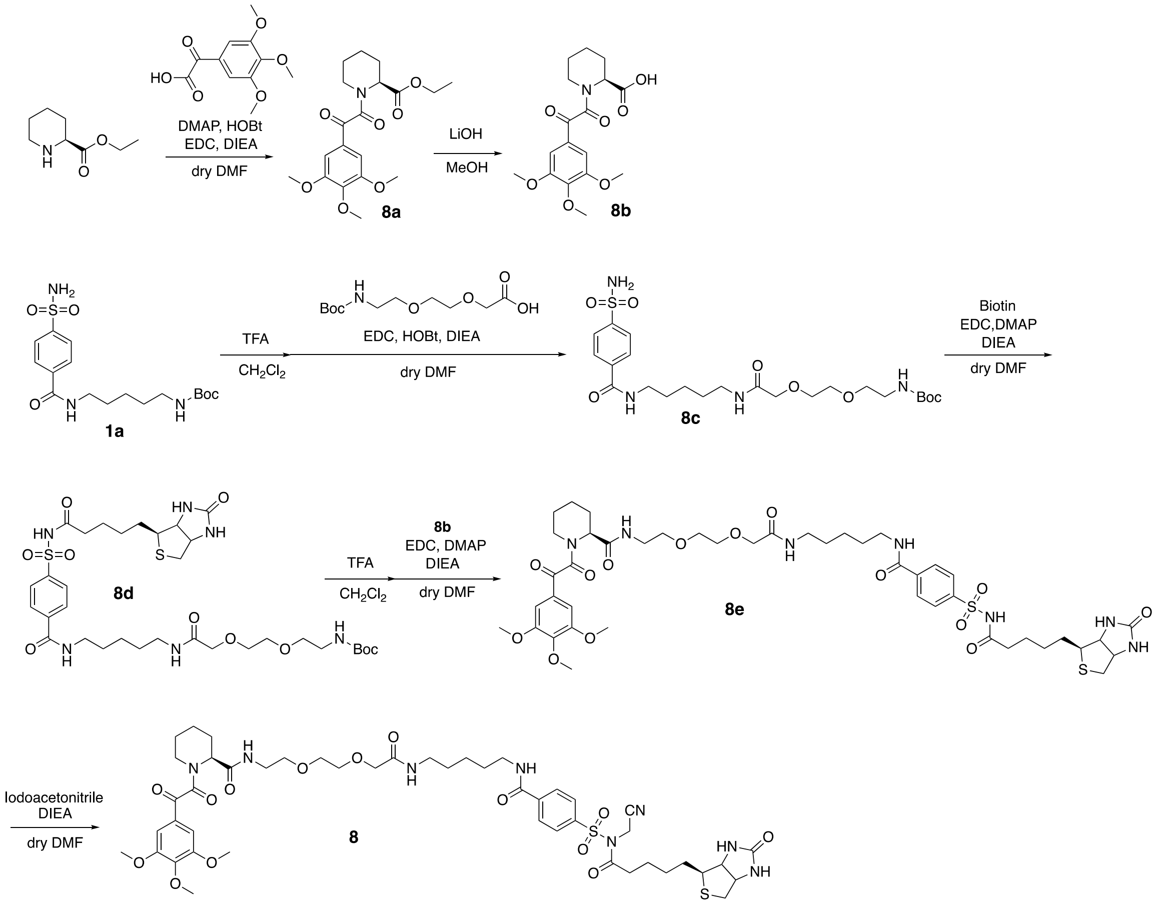

Supplementary Figure 26 Synthetic scheme of compound **8**

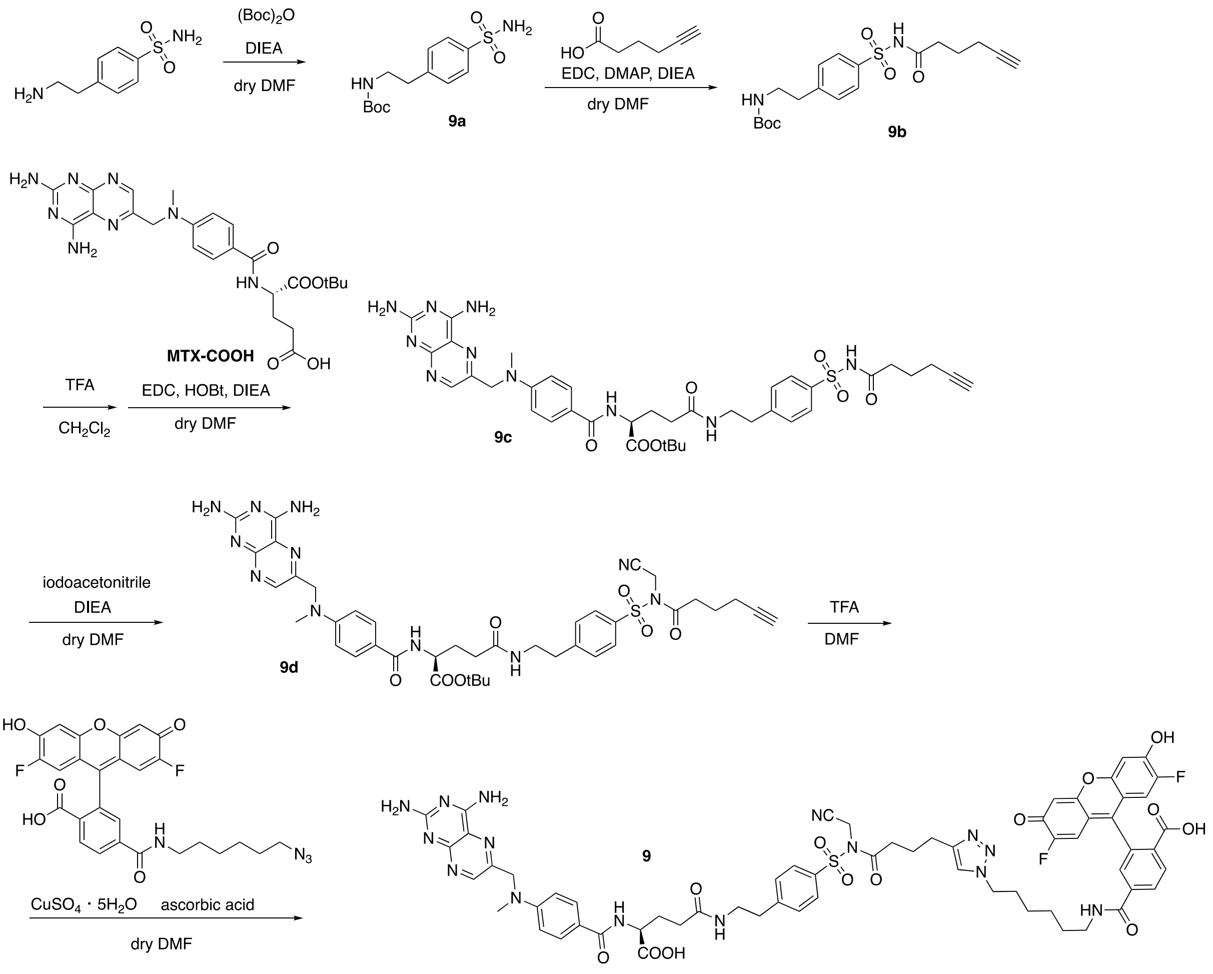

Supplementary Figure 27 Synthetic scheme of compound **9**

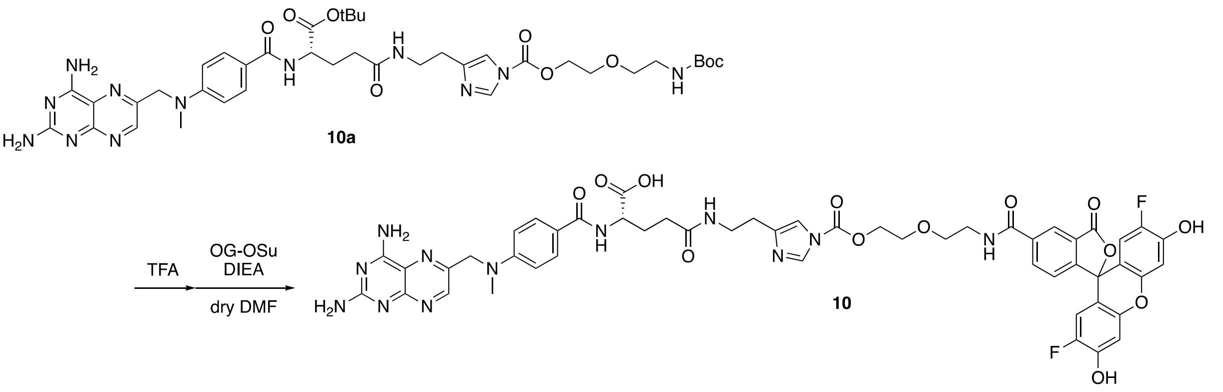

Supplementary Figure 28 Synthetic scheme of compound **10**

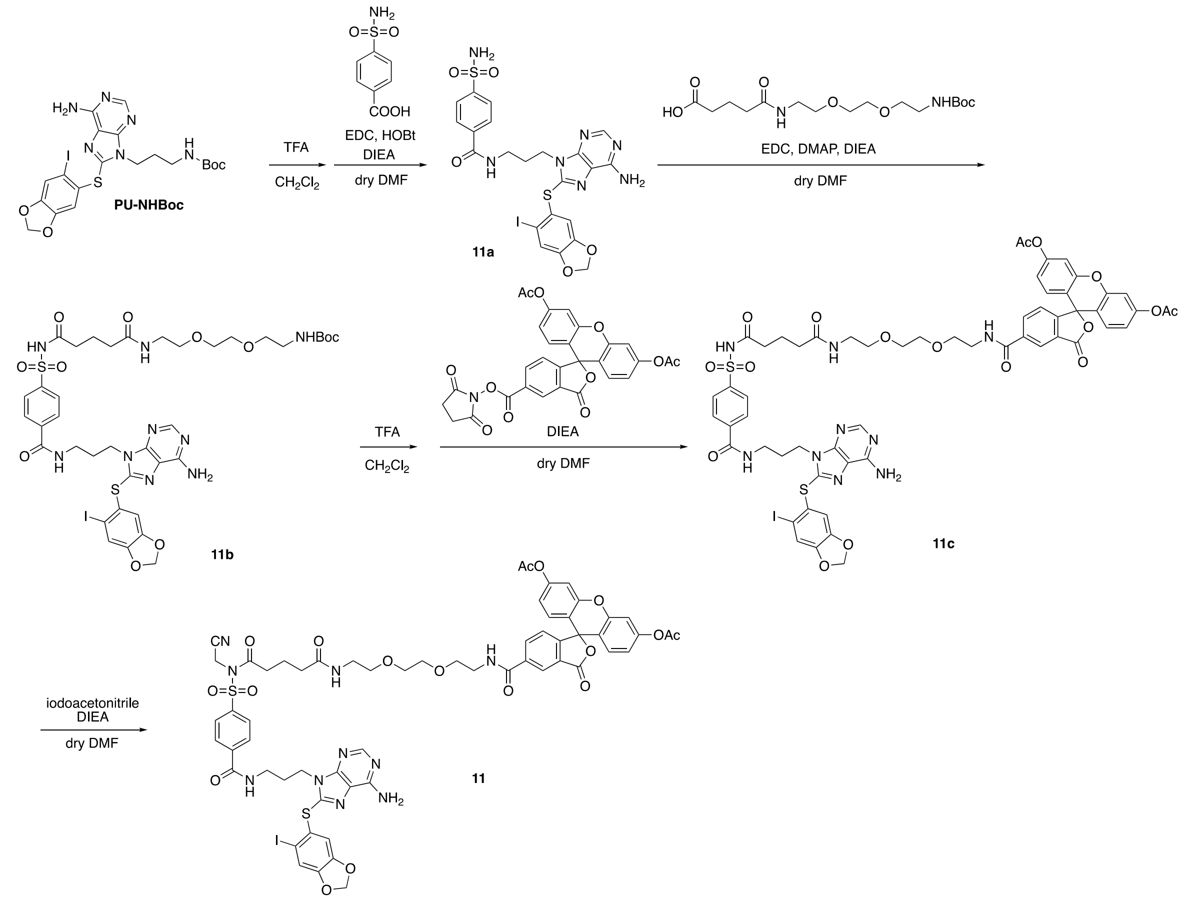

Supplementary Figure 29 Synthetic scheme of compound **11**

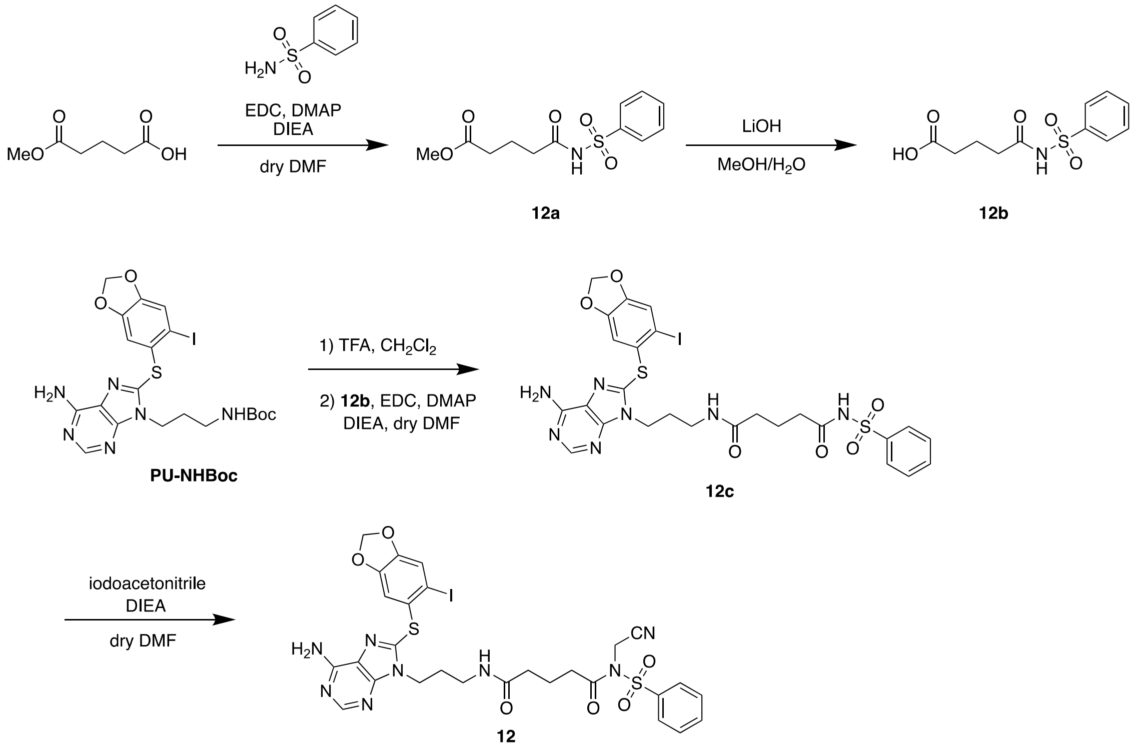

Supplementary Figure 30 Synthetic scheme of compound **12**

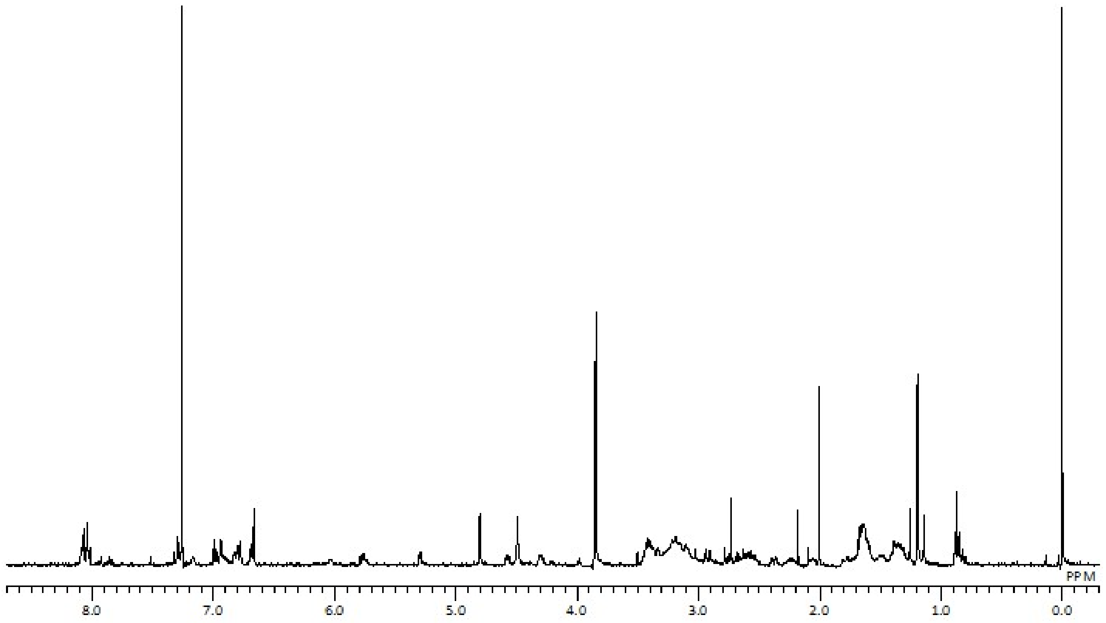

Supplementary Figure 31 ^{1}H-NMR spectrum of compound **1**.

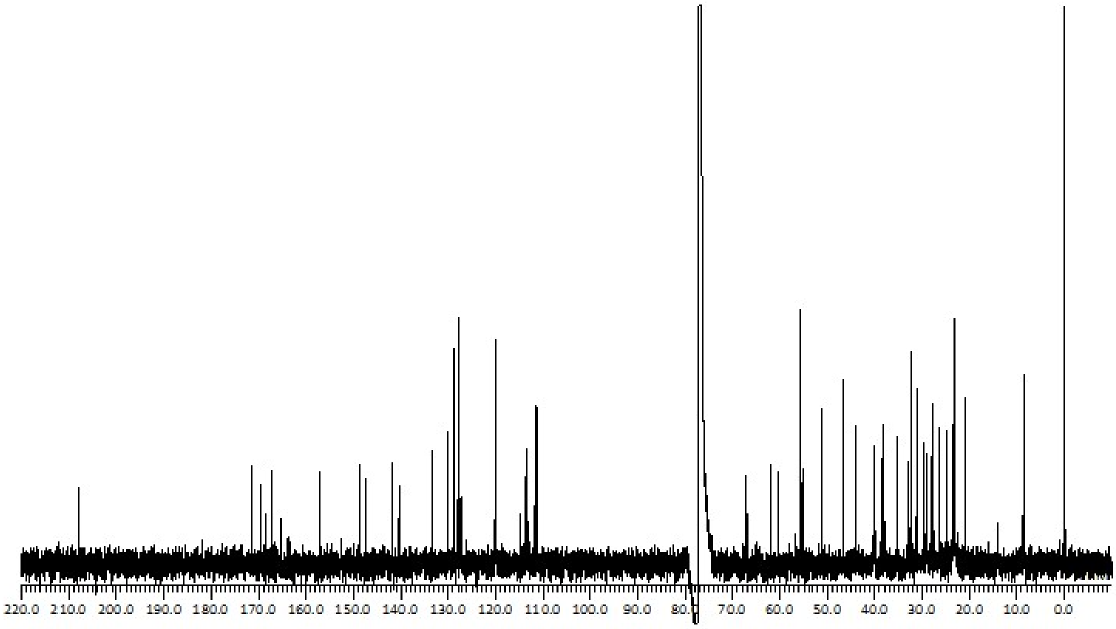

Supplementary Figure 32 ^{13}C-NMR spectrum of compound **1**.

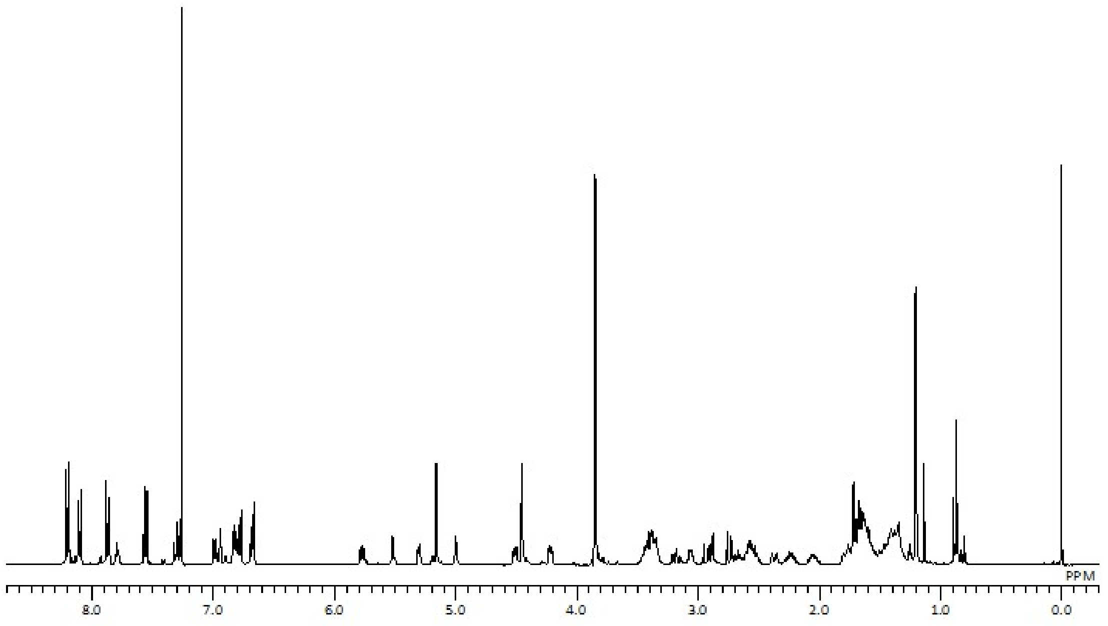

Supplementary Figure 33 ^{1}H-NMR spectrum of compound **2**.

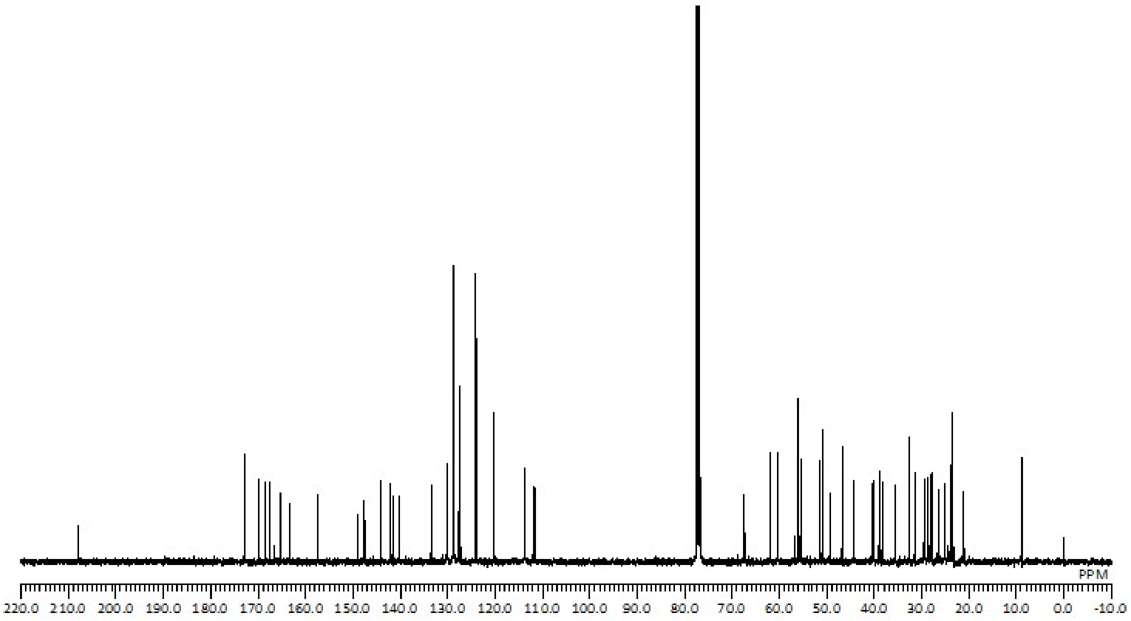

Supplementary Figure 34 ^{13}C-NMR spectrum of compound **2**.

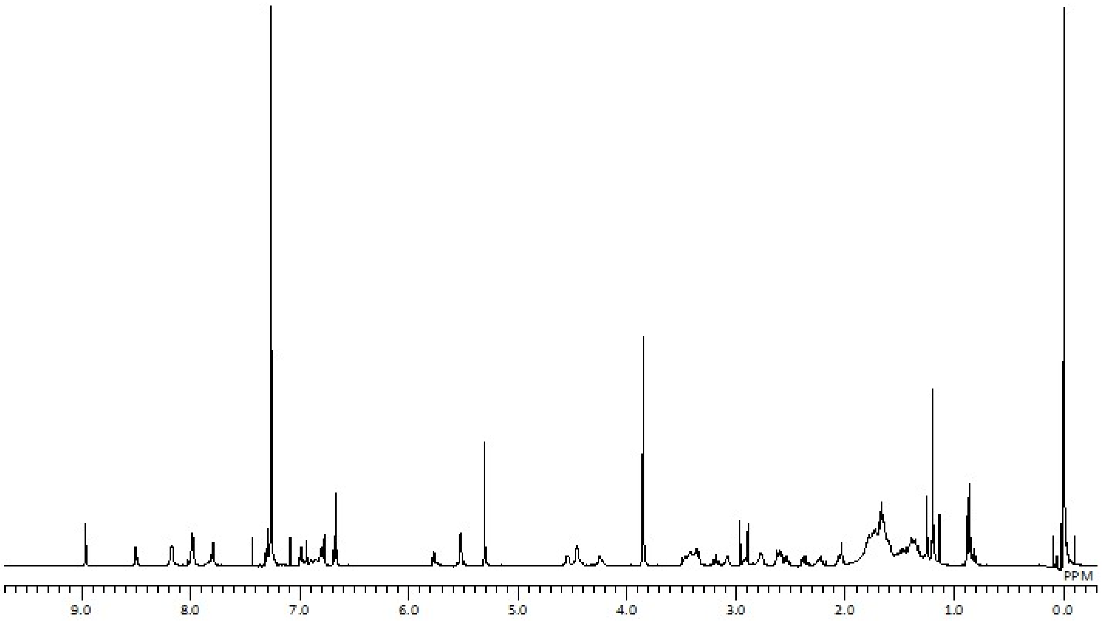

Supplementary Figure 35 ^{1}H-NMR spectrum of compound **3**.

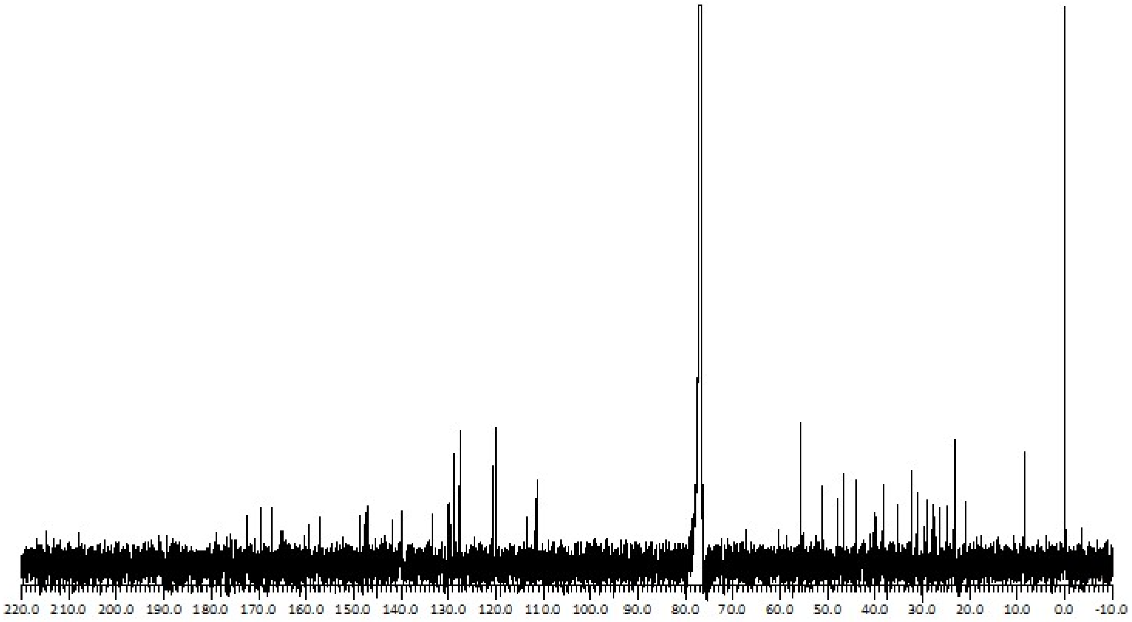

Supplementary Figure 36 ^{13}C-NMR spectrum of compound **3**.

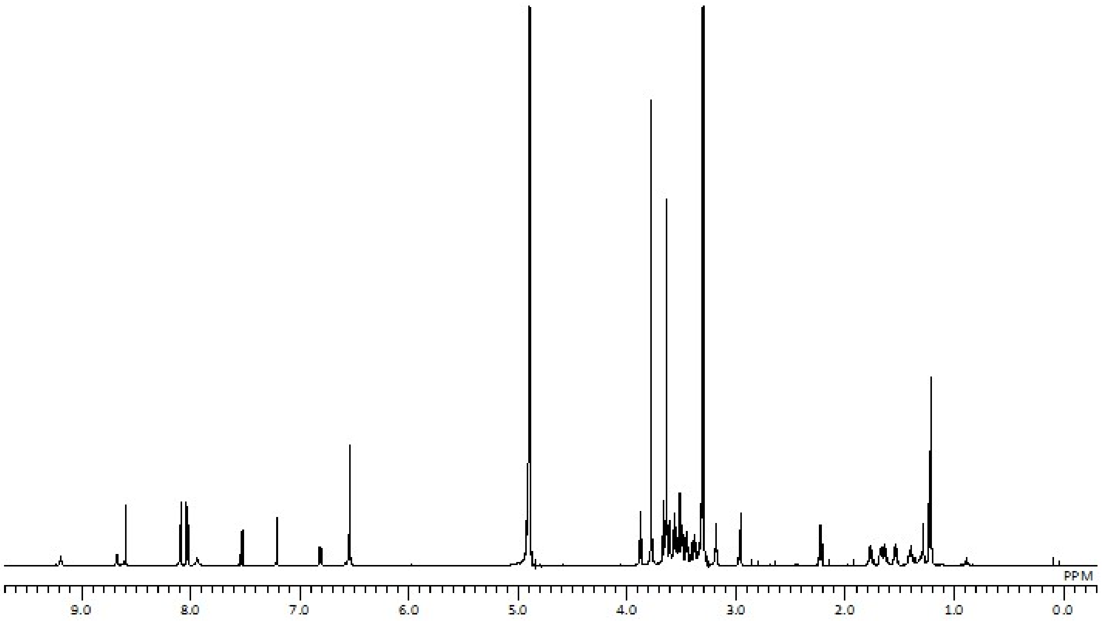

Supplementary Figure 37 ^{1}H-NMR spectrum of compound **4**.

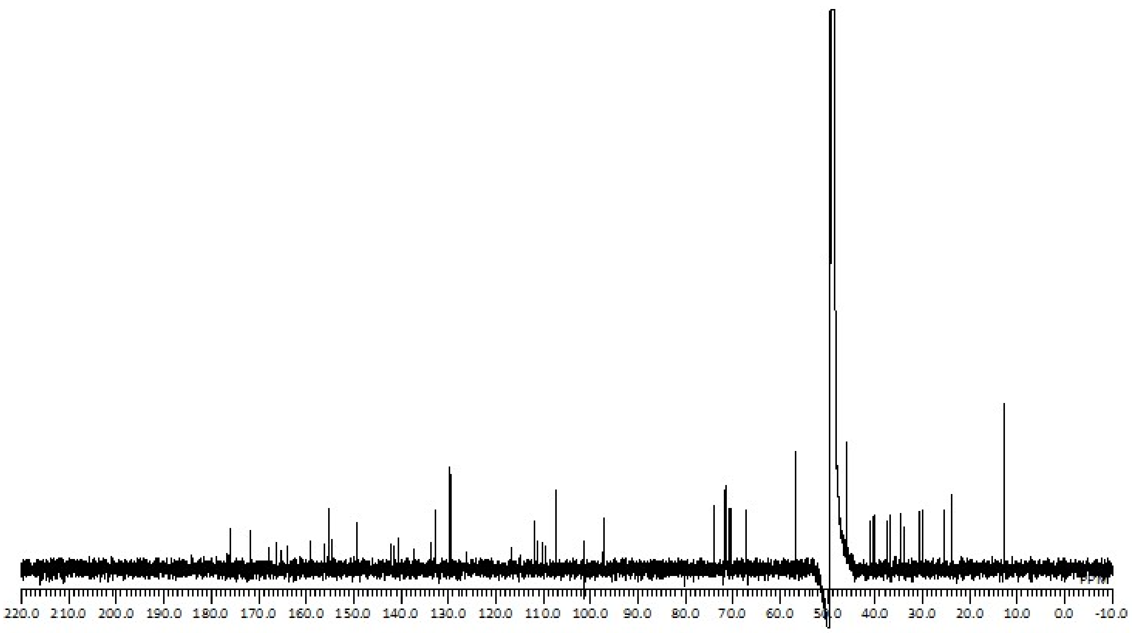

Supplementary Figure 38 ^{13}C-NMR spectrum of compound **4**.

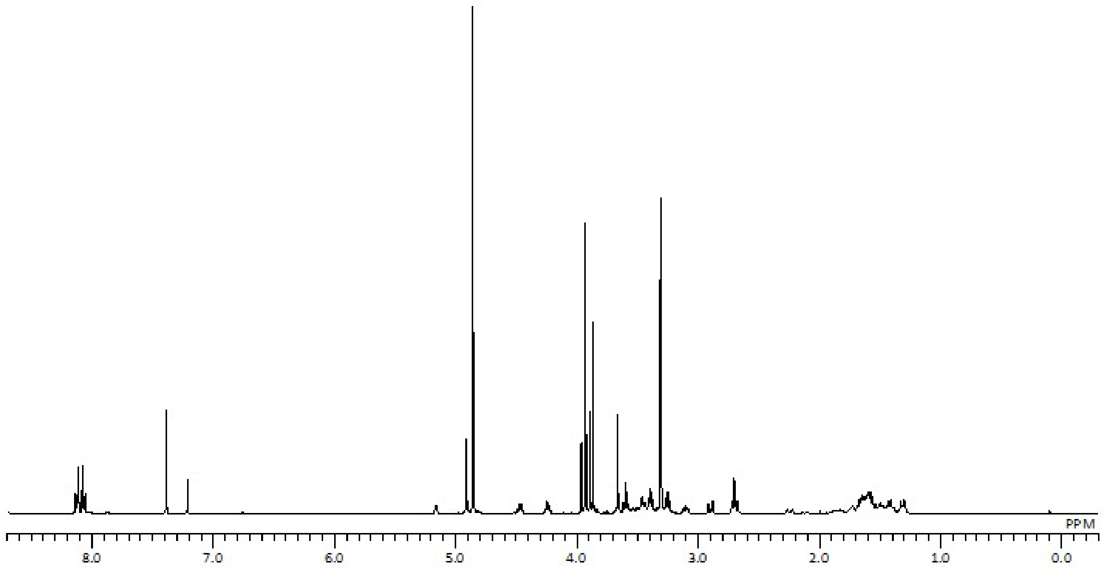

Supplementary Figure 39 ^{1}H-NMR spectrum of compound **8**.

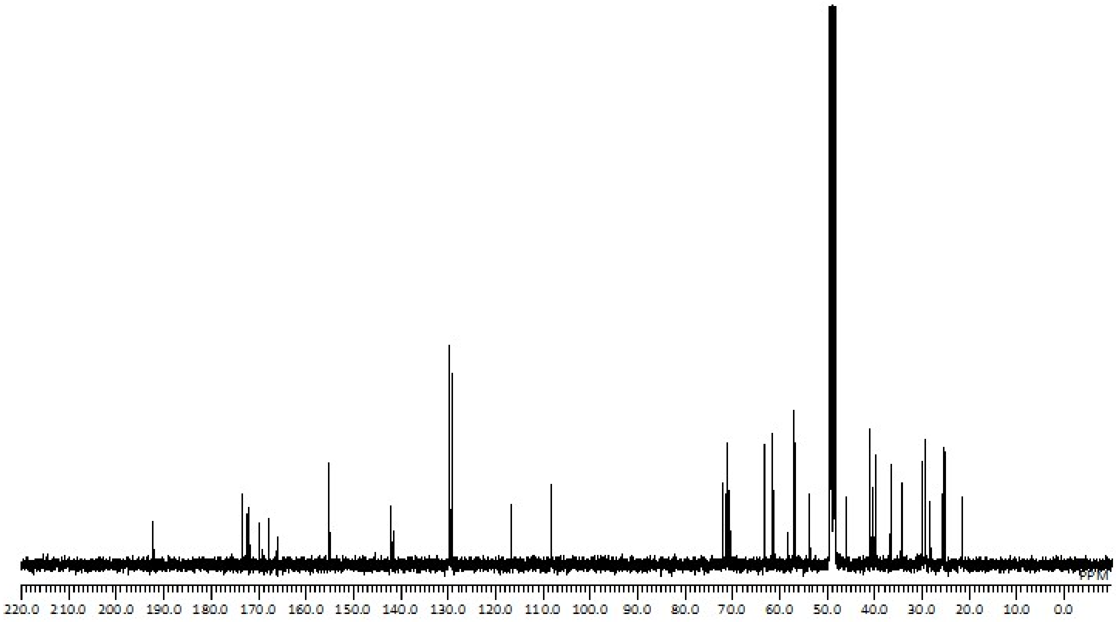

Supplementary Figure 40 ^{13}C-NMR spectrum of compound **8**.

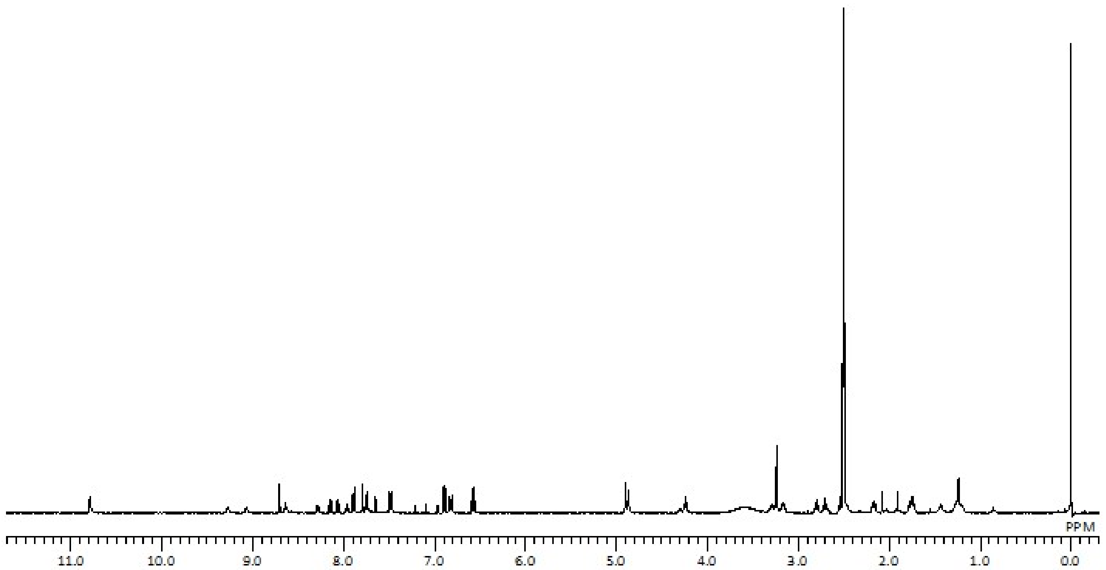

Supplementary Figure 41 ^{1}H-NMR spectrum of compound **9**.

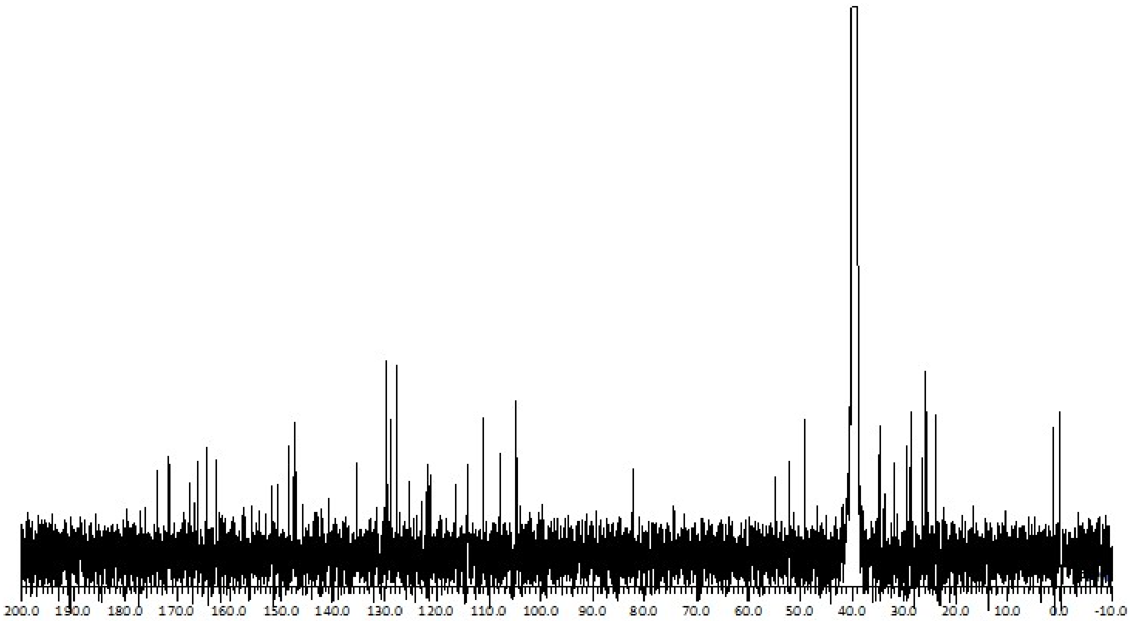

Supplementary Figure 42 ^{13}C-NMR spectrum of compound **9**.

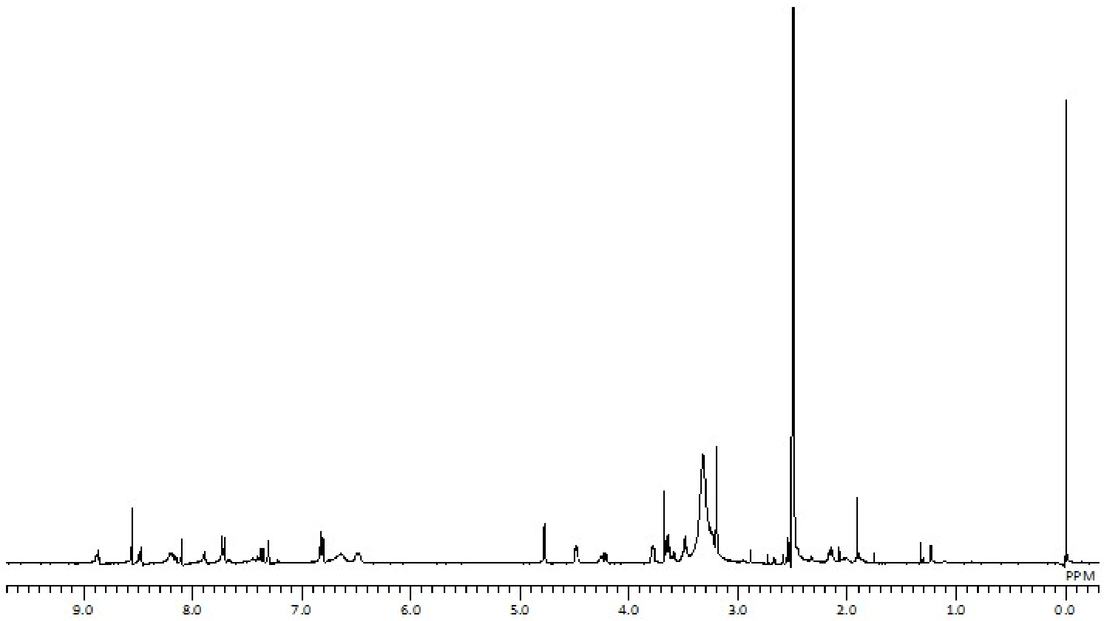

Supplementary Figure 43 ^{1}H-NMR spectrum of compound **10**.

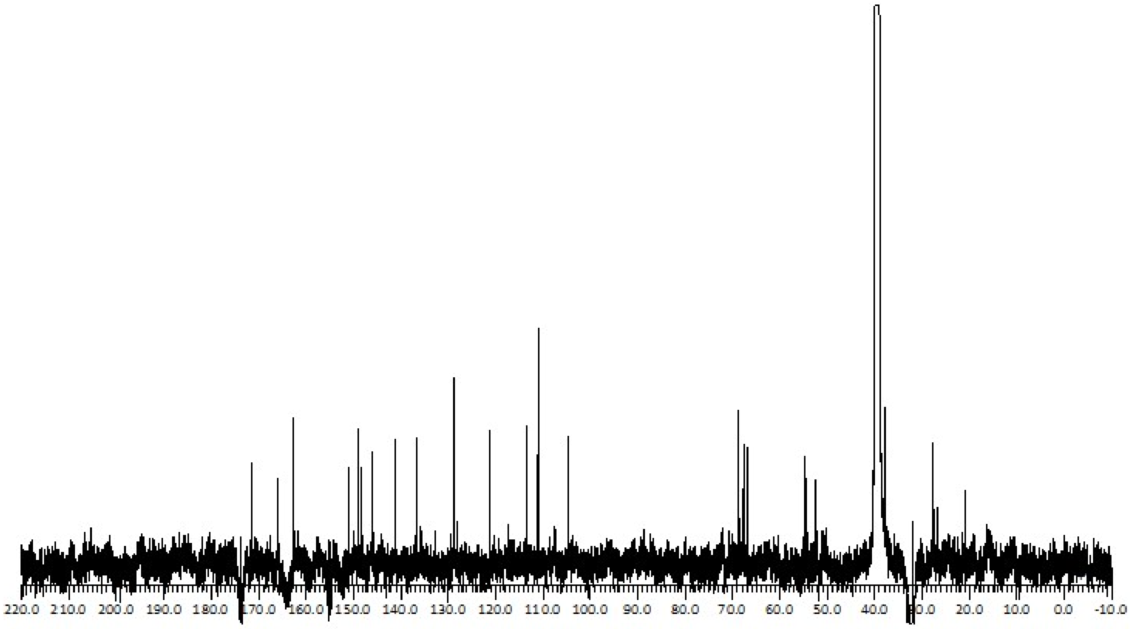

Supplementary Figure 44 ^{13}C-NMR spectrum of compound **10**.

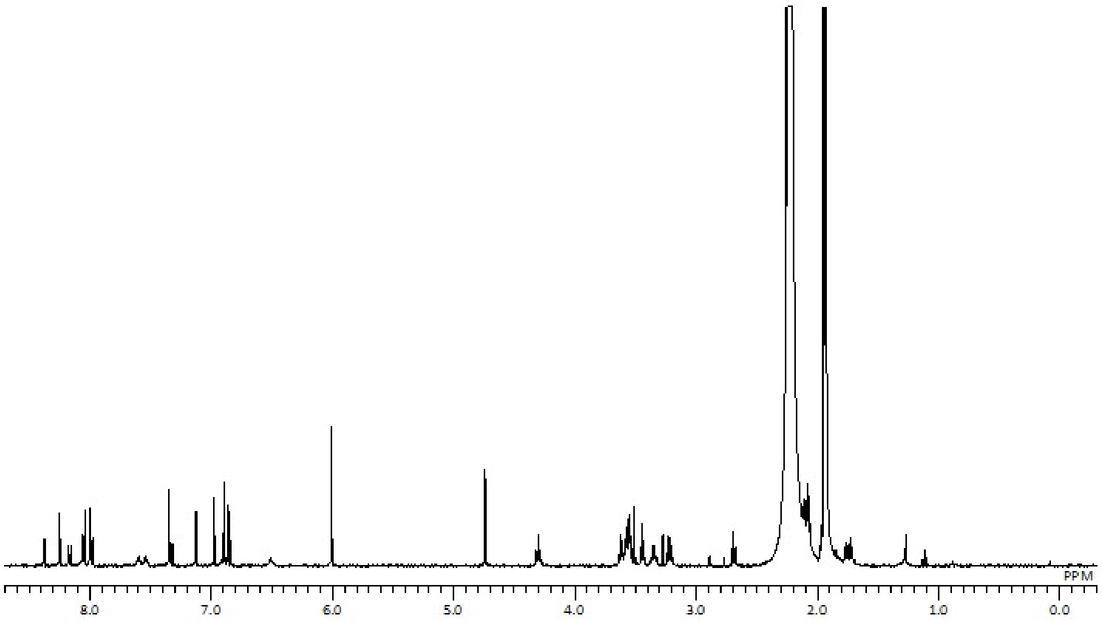

Supplementary Figure 45 ^{1}H-NMR spectrum of compound **11**.

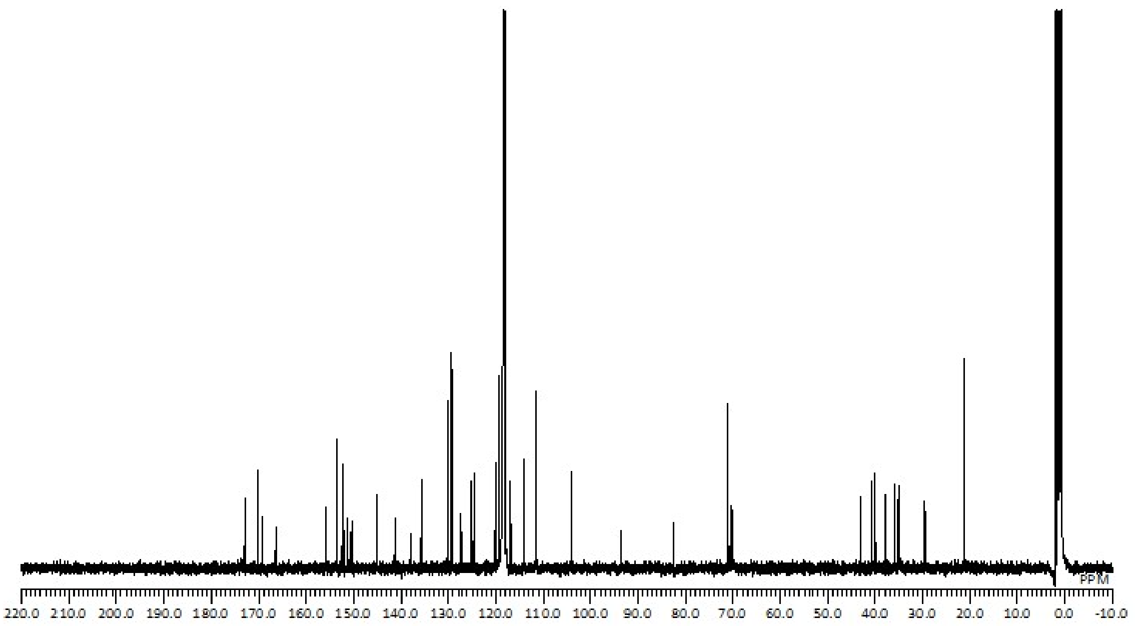

Supplementary Figure 46 ^{13}C-NMR spectrum of compound **11**.

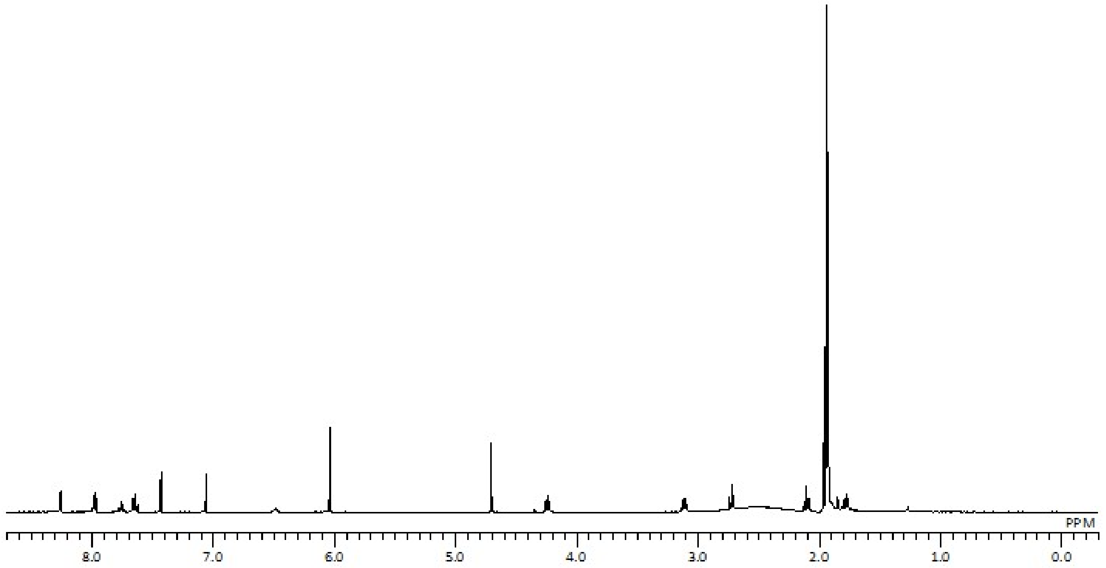

Supplementary Figure 47 ^{1}H-NMR spectrum of compound **12**.

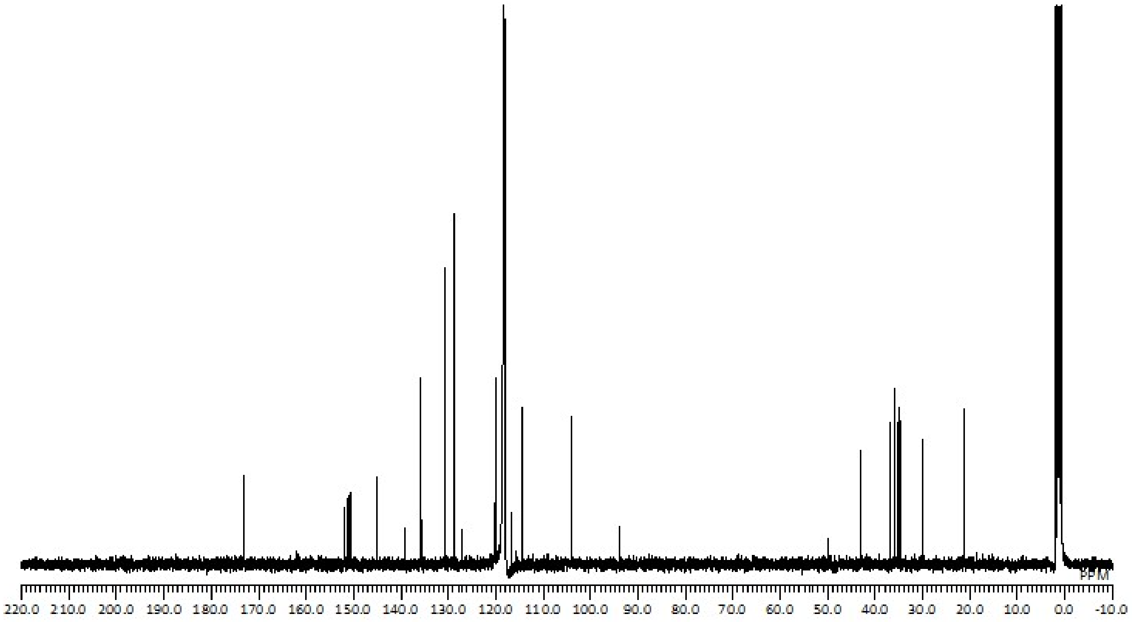

Supplementary Figure 48 ^{13}C-NMR spectrum of compound **12**.

Supplementary Methods

Kinetics of labelling reaction of LD chemistries

The labelling reaction of LD chemistry follows as Supplementary Equation (2)[3]:

$$P + R \underset{}{\overset{K_d}{\rightleftarrows}} PR \xrightarrow{k_L} P^* \qquad (2)$$

where P is a native (non-labelled) protein, R is a reagent, PR is a native protein-reagent complex, P* is a labelled-protein, K_d is a dissociation constant, and k_L is a rate constant for labelling process. Hydrolysis (deactivation) of LD reagents during the labelling process is negligible owing to these stabilities in buffer solution (Supplementary Figure 3, also see Supplementary References 4–6). The equations to be solved for the rate of labelling reaction are

$$[P]_0 = [P] + [PR] + [P^*] = [p] + [P^*] \quad ([p] = [P] + [PR])$$

$$K_d = \frac{[P][R]}{[PR]}$$

$$-\frac{d[p]}{dt} = k_L[PR]$$

The solution of the equations is

$$\ln\frac{[p]}{[P]_0} = -\frac{k_L t}{1 + K_d/[R]}$$

For $[R] >> [P]_0$, let

$$\frac{[P]+[PR]}{[P]_0} = \exp(-k_{app}t) \qquad (3)$$

$$k_{app} = \frac{k_L}{1 + K_d/[R]} \qquad (4)$$

According to the Supplementary Equation (4), the k_L and K_d values were obtained from the fitting analysis.

Preparation of purified FKBP12

Recombinant human FKBP12 was obtained as described previously.[4] Briefly, plasmide pGST-FXa-FKBP12 was transformed into *E. coli* BL21 Star (DE3) (Invitrogen). The cells were grown in LB media containing kanamycin at 37 °C to an optical density (660 nm) of 0.6, at which time the expression of the fusion protein was induced by the addition of 0.5 mM isopropyl β-D-1-thiogalactopyranoside (IPTG). After growth for an additional 6 h at 37 °C, the cells were harvested by centrifugation. The cell pellets were resuspended in 50 mM HEPES, 100 mM NaCl, 10% glycerol, pH 7.2, and lysed by sonication. The protein was purified from the soluble fraction of the lysate using a glutathione-Sepharose column chromatography (GE Healthcare) and dialyzed against 50 mM Tris, 100 mM NaCl, 5 mM $CaCl_2$, pH 8.0. The GST tag in the fusion protein was cleaved with factor Xa at 20 °C for 16 h. The resulting (tag-free) FKBP12 was purified by passing it through a benzamidine-Sepharose column (GE Healthcare), followed by a glutathione-Sepharose column. The concentration of FKBP12 was determined by measuring the absorbance at 280 nm using molar absorption coefficient of 9,860 $M^{-1}cm^{-1}$.

Preparation of purified eDHFR

Recombinant eDHFR was obtained as described previously.[7] Briefly, plasmid pBAD-DHFR (kindly gifted from Dr. Shinya Tsukiji) was transformed into *E. coli* strain Top10 (Invitrogen). The cells were grown in LB media containing ampicillin at 37 °C to an optical density (660 nm) of 0.6, at which time the expression of the fusion protein was induced by the addition of 0.1% L-arabinose and subsequent incubation for 16 h at 27 °C. The fusion proteins were purified using TALON metal affinity resin (Clontech) according to manufacturer’s instruction. After dialysis against PBS buffer, the purified proteins were incubated with thrombin (Novagen) at 20 °C for 24 h to eliminate the thioredoxin in the N-terminus of the fusion proteins. The resulting eDHFR was purified by passing it through a benzamidine-Sepharose column, followed by TALON metal affinity resin. The concentrations of eDHFR were determined using a BCA assay kit (Thermo).

Preparation of purified N-terminal domain of Hsp90α

N-terminal domain of Hsp90α was expressed and purified according to a slight modification of the method reported previously.[8] Plasmid GST-Hsp90N(9-236) purchased from Addgene were transformed into *E. coli* strain BL21 (DE3) and induced by 1 mM IPTG at an optical density (650 nm) of 0.8 for 20 h at 17 °C. The cultures were harvested and the cells were resuspended in a lysis buffer (50 mM tris-HCl, pH 8.0, 300 mM NaCl, 1 mM EDTA). Cells were disrupted by sonication and clarified by centrifugation. The protein was purified using a glutathione-Sepharose column chromatography and dialyzed against PBS buffer. The GST tag in the fusion protein was cleaved with thrombin at 20 °C for 16 h. The resulting (tag-free) N-terminal domain of Hsp90α was purified by passing it through a benzamidine-Sepharose column, followed by a glutathione-Sepharose column. The concentration of N-terminal domain of Hsp90α was determined using a BCA assay kit.

General materials and methods for organic synthesis

All chemical reagents and solvents were obtained from commercial suppliers (Sigma-Aldrich, Tokyo Chemical Industry (TCI), Wako Pure Chemical Industries, Sasaki Chemical, Nacalai tesque, Novabiochem or Watanabe Chemical Industries) and used without further purification. All reactions were carried out under an atmosphere of nitrogen unless otherwise noted. Thin layer chromatography (TLC) was performed on silica gel 60 F254 precoated aluminium sheets or glass plates (Merck) and visualized by fluorescence quenching or ninhydrin staining. Chromatographic purification was accomplished using flash column chromatography on silica gel 60 N (neutral, 40–50 μm, Kanto Chemical). ^{1}H- and ^{13}C-NMR spectra were recorded in deuterated solvents on a Varian Mercury 400 (400 MHz) spectrometer or JEOL JNM-ECA (600 MHz), and calibrated to the residual solvent peak or tetramethylsilane (= 0 ppm). Multiplicities are abbreviated as follows: s = singlet, d = doublet, t = triplet, q = quartet, m = multiplet, dd = double doublet. Matrix-assisted laser desorption/ionization time-of-flight mass spectrometry (MALDI-TOF MS) spectra were recorded on an Autoflex III instrument (Bruker Daltonics) using α-cyano-4-hydroxycinnamic acid (CHCA) as the matrix. High-resolution mass spectra were measured on an Exactive (Thermo Scientific) equipped with electron spray

ionization (ESI). Reversed-phase HPLC (RP-HPLC) was carried out on a Hitachi LaChrom L-7100 system equipped with a LaChrom L-7400 UV detector, and a YMC-Pack ODS-A column (5 μm, 250 × 20 mm) at a flow rate of 9.9 mL/min. UV detection was at 220 nm. All runs used linear gradients of acetonitrile containing 0.1% TFA (solvent A) and 0.1% aqueous TFA (solvent B).

Synthesis of compound 1

*Compound **1a***

To a stirred solution of 4-sulfamoylbenzoic acid (400 mg, 1.99 mmol) in dry *N*, *N*-dimethylformamide (DMF) (8 mL) was added *N*-(*tert*-Butoxycarbonyl)-1,5-diaminopentane hydrochloride (474 mg, 1.99 mmol), 1-ethyl-3-(3-dimethylaminopropyl)carbodiimide hydrochloride (EDC) (572 mg, 2.98 mmol), 1-hydroxybenzotriazole monohydrate (HOBt) (456 mg, 2.98 mmol) and *N*, *N*-diisopropylethylamine (DIEA) (1.04 mL, 5.97 mmol). The mixture was stirred overnight at room temperature. The solution was dissolved in EtOAc (100 mL), and washed with Sat. $NaHCO_3$ aq (30 mL×2), 5% citric acid (30 mL×1) and brine (30 mL×1). The organic layer was dried over $MgSO_4$, filtered and evaporated to yield compound **1a** (740 mg, 1.92 mmol, 96 %) as a colorless solid.

^{1}H NMR (400 MHz; CD_3OD): δ 7.98–7.93 (m, 4H), 3.39 (t, 2H, J = 7.2 Hz), 3.04 (t, 2H, J = 7.2 Hz), 1.66–1.62 (m, 2H), 1.54–1.49 (m, 2H), 1.41–1.37 (m, 11H).

*Compound **1b***

To a stirred solution of compound **1a** (200 mg, 0.519 mmol) in dry DMF (6 mL) was added biotin (210 mg, 0.855 mmol), EDC (300 mg, 1.56 mmol), 4-dimethylaminopyridine (DMAP) (21 mg, 0.172 mmol) and DIEA (270 μL, 1.55 mmol). The mixture was stirred overnight at room temperature. The solvent was removed under reduced pressure, and the residue was purified by flash chromatography on silica gel ($CHCl_3$: MeOH : AcOH = 150:10:1) to yield compound **1b** (135 mg, 0.221 mmol, 43 %) as a colorless solid

^{1}H NMR (400 MHz; CD_3OD): δ 8.07 (d, 2H, J = 8.4 Hz), 7.97 (d, 2H, J = 8.4 Hz), 4.47–4.44 (m, 1H), 4.22–4.19 (m, 1H), 3.41–3.37 (m, 2H), 3.10–3.02 (m, 3H), 2.90 (dd, 1H, J = 4.8, 12.8 Hz), 2.67 (d, 1H, J = 12.8 Hz), 2.24 (t, 2H, J = 7.2 Hz), 1.68–1.60 (m, 4H), 1.58–1.46 (m, 4H), 1.41–1.38 (m, 11H), 1.31–1.23 (m, 2H).

Compound ***1c***

To a stirred solution of compound **1b** (18 mg, 29 μmol) in CH_2Cl_2 (2 mL) was added trifluoroacetic acid (TFA) (1 mL) at room temperature. The reaction solution was allowed to stir for 30 min. TFA was removed by twice co-evaporation with toluene (1 mL). To a stirred solution of this residue in dry DMF (2 mL) was added **SLF**[9] (17 mg, 29 μmol), EDC (8.5 mg, 44 μmol), HOBt (6.7 mg, 44 μmol) and DIEA (25 μL, 147 μmol). The mixture was stirred overnight at room temperature. The solvent was removed under reduced pressure, and the residue was purified by flash chromatography on silica gel ($CHCl_3$: MeOH = 20:1) to yield compound **1c** (26 mg, 24 μmol, 82 %) as colorless oil.

^{1}H NMR (400 MHz; $CDCl_3$): δ 8.00 (d, 2H, *J* = 8.4 Hz), 7.89 (d, 2H, *J* = 8.4 Hz), 7.28–7.26 (m, 1H), 7.18 (bs, 1H), 6.97–6.90 (m, 2H), 6.81–6.76 (m, 2H), 6.69–6.66 (m, 2H), 6.12 (bs, 1H), 5.77–5.69 (m, 2H), 5.30 (d, 1H, *J* = 4.4 Hz), 4.53–4.50 (m, 1H), 4.46 (s, 2H), 4.23–4.20 (m, 1H), 3.84 (s, 3H), 3.83 (s, 3H), 3.42–3.34 (m, 5H), 3.20–3.14 (m, 1H), 3.05–3.01 (m, 1H), 2.86 (dd, 1H, *J* = 4.8, 12.8 Hz), 2.70 (d, 1H, *J* = 12.8 Hz), 2.59–2.52 (m, 2H), 2.41–2.35 (m, 2H), 2.24–2.19 (m, 1H), 2.06–2.00 (m, 1H), 1.78–1.28 (m, 14H), 1.20–1.12 (m, 6H), 0.86 (t, 3H, *J* = 7.6 Hz).

Compound ***1***

To a stirred solution of compound **1c** (13 mg, 12 μmol) in dry DMF (1 mL) was added iodoacetonitrile (8.7 μL, 0.12 mmol) and DIEA (10 μL, 60 μmol). The mixture was stirred overnight at room temperature. The solvent was removed under reduced pressure, and the residue was purified by flash chromatography on silica gel ($CHCl_3$: MeOH = 40:1→20:1→15:1) to yield compound **1** (27 mg, 65 μmol, 83 %) as colorless oil.

^{1}H NMR (400 MHz; $CDCl_3$): δ 8.07 (d, 2H, *J* = 8.4 Hz), 8.02 (d, 2H, *J* = 8.4 Hz), 7.32–7.26 (m, 1H), 6.99–6.97 (m, 1H), 6.94–6.88 (m, 1H), 6.83–6.77 (m, 2H), 6.70–6.66 (m, 2H), 6.02 (s, 1H), 5.78–5.70 (m, 1H), 5.30–5.28 (m, 1H), 4.80 (s, 2H), 4.59 (m, 1H), 4.49 (s, 2H), 4.31–4.28 (m, 1H), 3.85 (s, 3H), 3.84 (s, 3H), 3.43–3.02 (m, 12H), 2.94–2.49 (m, 6H), 2.39–2.02 (m, 4H), 1.81–1.59 (m, 6H), 1.49–1.29 (m, 6H), 1.20–1.12 (m, 6H), 0.87 (t, 3H, *J* = 7.6 Hz).

^{13}C NMR (150 MHz, $CDCl_3$): δ 207.9, 171.5, 169.5, 168.6, 167.2, 165.2, 163.8, 157.1, 148.7, 147.2, 141.9, 140.5, 140.1, 133.2, 130.0, 128.8 (2C), 127.6 (2C), 120.0 (2C), 120.0, 113.6, 113.3, 111.6, 111.2, 76.3, 67.0, 62.1, 60.4, 55.8, 55.7, 55.2, 51.2, 46.6, 44.0, 40.2, 40.0, 38.3, 38.1, 35.1, 33.0, 32.3, 31.1, 29.6, 29.0, 28.1, 27.6, 26.3, 24.8, 23.5, 23.4, 23.3, 23.1, 21.0, 8.6.

HRMS (ESI): Calcd for $(C_{56}H_{74}N_7O_{13}S_2)^+$ $[M+H]^+$ 1116.4781, found: 1116.4790.

Synthesis of compound 2

Compound ***2***

To a stirred solution of compound **1c** (8.5 mg, 7.9 μmol) in dry DMF (1 mL) was added 4-nitrobenzylbromide (8.7 μL, 0.12 mmol), potassium iodide (0.4 mg, 2.4 μmol) and DIEA (4.1 μL, 24 μmol). The mixture was stirred at room temperature for 1 h and then at 40 °C for 4 h. The solvent was removed under reduced pressure, and the residue was purified by flash chromatography on silica gel ($CHCl_3$: MeOH = 20:1) to yield compound **2** (3.7 mg, 3.1 μmol, 39 %) as a colorless solid.

^{1}H NMR (400 MHz; $CDCl_3$): δ 8.20 (d, 2H, *J* = 8.8 Hz), 8.10 (d, 2H, *J* = 8.8 Hz), 7.87 (d, 2H, *J* = 8.8 Hz), 7.79 (t, 1H, *J* = 5.6 Hz), 7.56 (d, 2H, *J* = 8.8 Hz), 7.30 (t, 1H, *J* = 7.6 Hz), 6.99–6.89 (m, 2H), 6.84–6.76 (m, 3H), 6.69–6.66 (m, 2H), 5.77 (dd, 1H, *J* = 8.0, 5.2 Hz), 5.52 (s, 1H), 5.30 (m, 1H), 5.16 (s, 2H), 5.00 (s, 1H), 4.51 (m, 1H), 4.45 (s, 2H), 4.22 (m, 1H), 3.85 (s, 3H), 3.84 (s, 3H), 3.46–3.34 (m, 5H), 3.18 (dt, 1H, *J* = 12.8, 2.4 Hz), 3.05 (m, 1H), 2.90 (dd, 1H, *J* = 12.8, 4.8 Hz), 2.75 (d, 1H, *J* = 12.8 Hz), 2.71–2.51 (m, 4H), 2.37 (m, 1H), 2.23 (m, 1H), 2.06 (m, 1H), 1.80–1.21 (m, 18H), 1.20 (s, 3H), 1.13 (s, 3H), 0.87 (t, 3H, *J* = 7.2 Hz).

^{13}C NMR (100 MHz, $CDCl_3$): δ 208.0, 172.7, 169.7, 168.4, 167.4, 165.4, 163.4, 157.4, 149.0, 147.6, 147.5, 144.1, 142.1, 141.4, 140.2, 133.4, 130.1, 128.8 (2C), 128.7, 127.6 (2C), 124.0 (2C), 120.3, 120.2, 113.8, 113.6, 111.9, 111.5, 76.5, 67.4, 61.9, 60.3, 56.0, 55.9, 55.4, 51.4, 50.9, 49.3, 46.8, 44.2, 40.5, 40.1, 38.7, 38.3, 35.5, 32.5, 31.3, 29.3, 28.5, 27.9, 27.7, 26.5, 25.0, 23.9, 23.8, 23.5, 23.3, 21.2, 8.8.

HRMS (ESI): Calcd for $(C_{61}H_{78}N_7O_{15}S_2)^+$ $[M+H]^+$: 1212.4997, found: 1212.4992.

Synthesis of compound 3

Compound ***3***

To a stirred solution of compound **1c** (13.2 mg, 12.2 μmol) in dry DMF (1 mL) was added 2,4-dinitrobenzylbromide (16 mg, 61 μmol), potassium iodide (2.0 mg, 12 μmol) and DIEA (6.4 μL, 36 μmol). The mixture was stirred at 40 °C for 16 h. The solvent was removed under reduced pressure, and the residue was purified by RP-HPLC (gradient (A:B) 0 min; 20:80, 60 min; 80:20). The fraction containing the target compound was collected and lyophilized to give compound **3** (1.2 mg, 0.95 μmol, 8%) as a colorless solid.

^{1}H NMR (600 MHz; $CDCl_3$): δ 8.96 (d, 1H, *J* = 2.4 Hz), 8.50 (d, 1H, *J* = 8.4 Hz), 8.17 (d, 2H, *J* = 7.8 Hz), 7.98 (d, 2H, *J* = 7.8 Hz), 7.79 (d, 1H, *J* = 8.4 Hz), 7.29 (t, 1H, *J* = 8.4 Hz), 6.99–

6.93 (m, 2H) 6.80–6.77 (m, 2H), 6.69–6.66 (m, 2H), 5.77 (dd, 1H, *J* = 7.8, 6.0 Hz), 5.52 (d, 2H, *J* = 4.8 Hz), 5.30 (s, 2H), 5.26–5.22 (m, 1H), 4.54 (s, 1H), 4.45 (s, 2H), 4.24 (m, 1H), 3.85 (s, 3H), 3.84 (s, 3H), 3.49–3.34 (m, 5H), 3.18 (dt, 1H, *J* = 13.2, 2.4 Hz), 3.07 (brs, 1H), 2.91 (m, 1H), 2.78–2.76 (m, 2H), 2.63–2.53 (m, 3H), 2.38 (m, 1H), 2.24 (m, 1H), 2.04 (m, 1H), 1.78–1.33 (m, 18H), 1.20 (s, 3H), 1.19 (s, 3H), 0.86 (t, 3H, *J* = 7.8 Hz).
^{13}C NMR (150 MHz, $CDCl_3$): δ 207.9, 172.4, 169.5, 167.2, 165.1, 164.8, 159.3, 157.1, 148.7, 147.6, 147.2, 147.0, 141.9, 139.9, 133.2, 130.0, 129.5, 128.8 (2C), 127.9, 127.4 (2C), 124.4, 120.6, 120.1, 120.0, 113.5, 113.4, 111.6, 111.1, 76.3, 67.0, 60.3, 58.7, 55.8, 55.7, 55.2, 52.4, 51.2, 47.9, 46.6, 44.0, 40.2, 39.9, 38.4, 38.1, 35.1, 32.3, 31.2, 29.6, 28.9, 27.7, 27.5, 26.3, 24.8, 23.5, 23.4, 23.3, 23.1, 21.0, 8.6.
HRMS (ESI): Calcd for $(C_{61}H_{77}N_8O_{17}S_2)^+$ $[M+H]^+$: 1257.4843, found: 1257.4835.

Synthesis of compound 4

Compound ***4a***

To a stirred solution of 4-sulfamoylbenzoic acid (400 mg, 1.99 mmol) in dry DMF (8 mL) was added *N*-carbobenzoxy-1,5-diaminopentane hydrochloride (543 mg, 1.99 mmol), EDC (572 mg, 2.98 mmol), HOBt (456 mg, 2.98 mmol) and DIEA (1.04 mL, 5.97 mmol). The mixture was stirred overnight at room temperature. The solution was dissolved in EtOAc (100 mL) and washed with Sat. $NaHCO_3$ aq (30 mL×2), 5% citric acid (30 mL×1) and brine (30 mL×1). The organic layer was dried over $MgSO_4$, filtered and evaporated to yield compound **4a** (748 mg, 1.78 mmol, 89 %) as a colorless solid.
^{1}H NMR (400 MHz; CD_3OD): δ 7.97–7.92 (m, 4H), 7.33–7.26 (m, 5H), 5.04 (s, 2H), 3.41–3.36 (m, 2H), 3.15–3.11 (m, 2H), 1.66–1.60 (m, 2H), 1.58–1.51 (m, 2H), 1.44–1.40 (m, 2H).

Compound ***4b***

To a stirred solution of compound **4a** (570 mg, 1.36 mmol) in dry DMF (6 mL) was added 15-(Boc-amino)-4,7,10,13-tetraoxapentadecanoic acid (500 mg, 1.36 mmol), EDC (391 mg, 2.04 mmol), DMAP (50 mg, 0.41 mmol) and DIEA (1.2 mL, 6.8 mmol). The mixture was stirred overnight at room temperature. The solution was dissolved in EtOAc (150 mL) and washed with Sat. $NaHCO_3$ aq (30 mL×1), 5% citric acid (30 mL×2) and brine (30 mL×1). The organic layer was dried over $MgSO_4$, filtered and evaporated to yield compound **4b** (928 mg, 1.21 mmol, 89 %) as brown oil.

^{1}H NMR (400 MHz; $CDCl_3$): δ 8.02 (d, 2H, *J* = 8.0 Hz), 7.77 (d, 2H, *J* = 8.0 Hz), 7.51–7.29 (m, 5H), 6.48 (s, 1H), 5.38 (s, 1H), 5.06 (s, 2H), 4.89 (s, 1H), 3.73 (t, 2H, *J* = 5.2 Hz), 3.64–3.60 (m, 10H), 3.54 (t, 2H, *J* = 4.8 Hz), 3.48–3.46 (m, 4H), 3.45–3.42 (m, 2H), 3.32–3.28 (m, 2H), 3.23–3.18 (m, 2H), 2.50 (t, 2H, *J* = 5.6 Hz), 1.64–1.52 (m, 4H), 1.45–1.41 (m, 11H).

Compound ***4c***

To a stirred solution of compound **4b** (111 mg, 145 μmol) in CH_2Cl_2 (3 mL) was added trifluoroacetic acid (TFA) (1 mL) at room temperature. The reaction solution was allowed to stir for 1 h. TFA was removed by twice co-evaporation with toluene (1 mL). To a stirred solution of this residue in dry DMF (2 mL) was added 7-diethylaminocoumarin-3-carboxylic acid (38 mg, 145 μmol), EDC (42 mg, 218 μmol), HOBt (34 mg, 218 μmol) and DIEA (126 μL, 725 μmol). The mixture was stirred overnight at room temperature. The solvent was removed under reduced pressure, and the residue was purified by flash chromatography on silica gel ($CHCl_3$: MeOH = 25:1) to yield compound **4c** (26 mg, 28 μmol, 19 %) as yellow oil.

^{1}H NMR (400 MHz; $CDCl_3$): δ 8.70 (s, 1H), 8.09 (d, 2H, *J* = 8.4 Hz), 7.89 (d, 2H, *J* = 8.4 Hz), 7.43 (d, 1H, *J* = 8.8 Hz), 7.32–7.25 (m, 4H), 6.66 (dd, 1H, *J* = 9.2, 2.4 Hz), 6.47 (d, 1H, *J* = 2.4 Hz), 5.05 (s, 2H), 3.75–3.72 (m, 18 H), 3.61 (t, 2H, *J* = 4.4 Hz), 3.46–3.42 (m, 6H), 3.22–3.18 (m, 2H), 2.52 (t, 2H, *J* = 5.6 Hz), 1.73 (s, 6H), 1.59–1.52 (m, 2H), 1.42–1.38 (m, 2H), 1.24 (t, 6H, *J* = 7.2 Hz).

Compound ***4d***

To a stirred solution of compound **4c** (26 mg, 28 μmol) in MeOH (5 mL) was added palladium on carbon (Pd/C) (10%, 10 mg) at room temperature. The reaction solution was allowed to stir overnight under H_2 atmosphere. The mixture was filtered and evaporated, and the residue was dissolved in dry DMF (2 mL). To this solution was added **TMP-COOH**[10] (10 mg, 28 μmol), EDC (8.1 mg, 42 μmol), HOBt (6.5 mg, 42 μmol) and DIEA (25 μL, 0.14 mmol). The mixture was stirred overnight at room temperature. The solvent was removed under reduced pressure, and the residue was purified by RP-HPLC (gradient (A:B) 0 min; 40:60, 10 min; 40:60, 50 min; 80:20) to yield compound **4d** (15 mg, 13 μmol, 46 %) as yellow oil.

^{1}H NMR (400 MHz; CD_3OD): δ 8.60 (s, 1H), 8.03 (d, 2H, *J* = 8.4 Hz) 7.94 (d, 2H, *J* = 8.4 Hz), 7.52 (d, 1H, *J* = 9.2 Hz), 7.22 (s, 1H), 6.80 (dd, 1H, *J* = 9.2, 2.2 Hz), 6.54 (s, 3H), 3.88 (t, 2H, *J* = 6.0 Hz), 3.77 (s, 6H), 3.64–3.44 (m, 26H), 3.37 (t, 2H, *J* = 7.2 Hz), 3.18 (t, 2H, *J* =

6.8 Hz), 2.46 (t, 2H, J = 6.8 Hz), 2.23 (t, 2H, J = 7.4 Hz), 1.76–1.73 (m, 2H), 1.69–1.61 (m, 4H), 1.56–1.52 (m, 2H), 1.45–1.30 (m, 2H), 1.23 (t, 6H, J = 7.2 Hz).

Compound ***4***

To a stirred solution of compound **4d** (11.4 mg, 10.1 µmol) in dry DMF (1.5 mL) was added iodoacetonitrile (7.4 µL, 101 µmol) and DIEA (8.8 µL, 50 µmol). The mixture was stirred at room temperature overnight. The solvent was removed under reduced pressure, and the residue was purified by RP-HPLC (gradient (A:B) 0 min; 40:60, 10 min; 40:60, 50 min; 80:20) to yield compound **4** (2.1 mg, 1.8 µmol, 18%) as yellow oil.

^{1}H NMR (600 MHz; CD_3OD): δ 9.19 (t, 1H, J = 5.4 Hz), 8.67 (t, 1H, J = 5.4 Hz), 8.60 (s, 1H), 8.09 (d, 2H, J = 8.4 Hz), 8.03 (d, 2H, J = 8.4 Hz), 7.94 (t, 1H, J = 5.4 Hz), 7.53 (d, 1H, J = 9.0 Hz), 7.21 (s, 1H), 6.81 (dd, 1H, J = 9.0 Hz), 6.55–6.53 (m, 3H), 4.88 (s, 2H), 3.87 (t, 2H, J = 6.0 Hz), 3.77 (s, 6H), 3.66–3.60 (m, 13H), 3.58–3.48 (m, 11H), 3.46–3.36 (m, 6H), 3.20–3.16 (m, 2H), 2.96 (t, 2H, J = 6.0 Hz), 2.22 (t, 2H, J = 7.8 Hz), 1.77 (quint, 2H, J = 7.8 Hz), 1.69–1.61 (m, 4H), 1.54 (quint, 2H, J = 7.8 Hz), 1.42–1.37 (m, 2H), 1.23 (t, 6H, J = 7.2 Hz).

^{13}C NMR (150 MHz, CD_3OD): δ 176.0, 171.9, 167.9, 166.2, 165.4, 163.9, 159.2, 156.1, 155.1, 154.6, 149.3, 142.0, 141.4, 140.4, 137.1, 133.6, 132.6, 129.6 (2C), 129.3 (2C), 126.2, 116.6, 111.6, 111.1, 110.0, 109.4, 107.2, 101.3, 97.2, 73.9, 71.6, 71.5, 71.5, 71.5, 71.4, 71.3, 70.5, 67.1, 56.6 (2C), 49.5 (2C), 45.9 (2C), 41.0, 40.5, 40.1, 37.6, 36.8, 34.5, 33.9, 30.5, 30.1, 29.9, 25.2, 23.7, 12.7.

HRMS (ESI): Calcd for $(C_{57}H_{77}N_{10}O_{15}S)^+$ $[M+H]^+$: 1173.5291, found: 1173.5270.

Synthesis of compound 8

Compound **8a**

To a stirred solution of (*S*)-piperidine-2-carboxylic acid ethyl ester (18.0 mg, 0.115 mmol) in dry DMF (2 mL) was added oxo(3,4,5-trimethoxyohenyl)acetic acid (27.5 mg, 0.115 mmol), EDC (33.0 mg, 0.172 mmol), DMAP (4.2 mg, 0.034 mmol), HOBt (23.0 mg, 0.172 mmol) and DIEA (64 µL, 0.34 mmol). The mixture was stirred for 12 h at room temperature. The solvent was removed under reduced pressure, and the residue was dissolved in EtOAc (30 mL), washed with sat. $NaHCO_3$ aq. (10 mL×2), citric acid (10 mL×3) and brine (10 mL). The organic layer was dried over $MgSO_4$, filtered and evaporated to yield compound **8a** (23 mg, 61 µmol, 53 %).

^{1}H NMR (400 MHz, $CDCl_3$) δ 7.37 (s, 2H), 5.37 (m, 1H), 4.26 (q, 2H, J = 7.2 Hz), 3.96 (m,

9H), 3.49 (m, 1H), 3.27 (dt, 1H, *J* = 13, 3.5 Hz), 2.38 (d, 1H, *J* = 14.8 Hz), 1.84–1.75 (m, 3H), 1.67–1.53 (m, 2H), 1.34–1.31 (t, 3H, *J* = 7.2 Hz).

Compound **8b**

To a stirred solution of **8a** (23.0 mg, 61.6 µmol) in MeOH (1 mL) was added 1N LiOH aq. (133 µL, 133 µmol) on ice, and the reaction mixture was stirred for 4.5 h at room temperature. The pH of the reaction mixture was adjusted to pH 3–4 with 1N HCl aq.. The solution was extracted with $CHCl_3$ (10 mL×3), and the organic layer was dried over $MgSO_4$, filtered and evaporated to yield compound **8b** (16 mg, 46 µmol, 75 %) as a colorless solid.

^{1}H NMR (400 MHz, $CDCl_3$) δ 7.31 (s, 2H), 5.47–5.46 (m, 1H), 3.95 (s, 3H), 3.91 (s, 6H), 3.52 (m, 1H), 3.24 (dt, 1H, *J* = 12.8, 2.9 Hz), 2.42 (d, 1H, *J* = 14.8 Hz), 1.87–1.41 (m, 5H).

Compound **8c**

To a stirred solution of **1a** (300 mg, 0.778 mmol) in CH_2Cl_2 (10 mL) was added TFA (1 mL) at room temperature. The reaction solution was allowed to stir for 30 min. TFA was removed by twice co-evaporation with toluene (1 mL). To a stirred solution of the deprotected compound (113.6 mg, 0.398 mmol) in dry DMF (3 mL) was added 2-[2-(Boc-amino)ethoxy]ethoxyacetic acid (dicyclohexylammonium) salt (177 mg, 0.398 mmol), EDC (114 mg, 0.597 mmol), HOBt (91.3 mg, 0.597 mmol) and DIEA (410 µL, 2.39 mmol). The mixture was stirred for 13 h at room temperature. The solvent was removed under reduced pressure, and the residue was purified by flash chromatography on silica gel ($CHCl_3$: MeOH = 15 : 1) to yield compound **8c** (198 mg, 0.373 mmol, 94 %).

^{1}H NMR (400 MHz, CD_3OD) δ 7.99–7.94 (m, 4H), 3.96 (s, 2H) 3.66–3.62 (m, 4H), 3.53 (t, 2H, *J* = 5.8 Hz), 3.40 (t, 2H, *J* = 7.0 Hz), 3.27–3.22 (m, 4H), 1.69–1.58 (m, 4H), 1.47–39 (m, 11H).

Compound **8d**

To a stirred solution of compound **8c** (100 mg, 0.188 mmol) in dry DMF (3 mL) was added biotin (50.6 mg, 0.207 mmol), EDC (72 mg, 0.376 mmol), DMAP (23.4 mg, 0.188 mmol) and DIEA (100 µL, 0.564 mmol). The mixture was stirred for 12 h at room temperature. The solvent was removed under reduced pressure, and the residue was purified by flash chromatography on silica gel ($CHCl_3$: MeOH : AcOH = 200 : 20 : 1) to yield compound **8d** (148.5 mg, 0.196 mmol, quant.).

^{1}H NMR (400 MHz, CD_3OD) δ 8.07 (d, 2H, *J* = 8.8 Hz), 7.97 (d, 2H, *J* = 8.8 Hz), 4.48–4.45 (m, 1H), 4.23–4.20 (m, 1H), 3.96 (s, 2H), 3.66–3.62 (m, 4H), 3.52 (t, 2H, *J* = 5.6 Hz), 3.40 (t,

2H, *J* = 6.8 Hz), 3.25–3.22 (m, 4H), 3.11–3.07 (m, 1H), 2.89 (dd, 1H, *J* = 12.8, 4.8 Hz), 2.68 (d, 1H, *J* = 12.8 Hz), 2.24 (t, 2H, *J* = 7.0 Hz), 1.71–1.49 (m, 10H), 1.43 (s, 9H), 1.35–1.26 (m, 2H).

Compound **8e**

To a stirred solution of **8d** (19 mg, 25 μmol) in CH_2Cl_2 (1 mL) was added TFA (0.5 mL) at room temperature. The reaction solution was allowed to stir for 30 min. TFA was removed by twice co-evaporation with toluene (1 mL). To a stirred solution of this residue in dry DMF (3 mL) was added compound **8b** (8.0 mg, 23 μmol), EDC (8.6 mg, 0.60 mmol), DMAP (3.0 mg, 22 μmol) and DIEA (24 μL, 0.13 mmol). The mixture was stirred for 13 h at room temperature. The solvent was removed under reduced pressure, and the residue was purified by flash chromatography on silica gel ($CHCl_3$: MeOH : AcOH = 200 : 20 : 1) to yield compound **8e** (5.0 mg, 5.1 μmol, 23%).

^{1}H NMR (400 MHz, CD_3OD) δ 8.05 (d, 2H, *J* = 8.4 Hz), 7.95 (d, 2H, *J* = 8.4 Hz), 7.39 (s, 2H), 5.17–5.16 (m, 1H), 4.47–4.44 (m, 1H) 4.22–4.19 (m, 1H), 3.96–3.86 (m, 11H), 3.68–3.35 (m, 12H), 3.28–3.23 (m, 4H), 3.10–3.06 (m, 1H), 2.89 (dd, 1H, *J* = 12.8, 5.0 Hz), 2.67 (d, 1H, *J* = 12.8 Hz), 2.23 (t, 2H, *J* = 7.2 Hz) 1.66–1.29 (m, 16H).

Compound **8**

To a stirred solution of compound **8e** (5.0 mg, 5.1 μmol) in dry DMF (0.5 mL) was added iodoacetonitrile (1.5 μL, 20 μmol) and DIEA (7.0 μL, 40 μmol). The mixture was stirred for 13 h at room temperature. The solvent was removed under reduced pressure, and the residue was purified by RP-HPLC (gradient (A:B) 0 min; 20:80, 40 min; 60:40) to yield compound **8** (2.0 mg, 1.9 μmol, 38 %).

^{1}H NMR (400 MHz, CD_3OD): δ 8.14–8.06 (m, 4H), 7.39 (s, 1.5H), 7.21 (s, 0.5H) 5.17–5.16 (m, 1H), 4.91 (s, 2H), 4.48–4.45 (m, 1H), 4.26–4.22 (m, 1H), 3.96–3.87 (m, 11H), 3.67–3.56 (m, 6H), 3.49–3.35 (m, 6H), 3.27–3.23 (m, 2H), 3.13– 3.08 (m, 1H), 2.92–2.88 (dd, 1H, *J* = 12.8, 4.8 Hz), 2.72–2.67 (m, 3H), 2.25 (brd, 0.7H, *J* = 14.4 Hz), 2.12 (brd, 0.3H, *J* = 13.2 Hz), 1.67–1.28 (m, 18H).

^{13}C NMR (100 MHz, CD_3OD): δ 192.2, 173.4, 172.5, 172.2, 169.9, 137.9, 166.0, 155.0, 154.8, 142.2, 141.6, 129.7 (2C), 129.4, 129.2, 116.7, 108.2, 72.0, 71.3, 71.1, 70.6, 63.3, 61.6, 61.2, 57.0, 56.9, 56.8, 53.6, 49.2, 49.0, 48.8, 48.4, 46.0, 41.0, 40.4, 39.7, 36.6, 34.4, 30.1, 29.9, 29.3, 29.2, 28.2, 25.8, 25.2, 21.4.

HRMS (ESI) calculated for $C_{47}H_{65}N_8O_{14}S_2$ ($[M+H]^+$) 1029.4056, found 1029.4047.

Synthesis of compound 9

Compound **9a**

To a stirred solution of *p*-(2-aminoethyl)benzenesulfonamide (1.000 g, 4.993 mmol) in dry DMF (20 mL) was added di-*tert*-butyl-dicarbonate (1.199 g, 5.492 mmol) and DIEA (1.3 mL, 7.5 mmol). The mixture was stirred for 30 min at room temperature. The solvent was removed under reduced pressure, and the residue was washed with $CHCl_3$ to yield compound **9a** (854.9 mg, 2.846 mmol, 57 %).

^{1}H NMR (400 MHz, DMSO-d_6): δ 7.73 (d, 2H, J = 8.4 Hz), 7.37 (d, 2H, J = 8.4 Hz), 7.28 (brs, 2H), 6.91 (brs, 1H), 3.16 (m, 2H), 2.76 (t, 2H, J = 7.2 Hz), 1.36 (s, 9H).

Compound **9b**

To a stirred solution of **9a** (500 mg, 1.67 mmol) in dry DMF (15 mL) was added 5-hexynoic acid (224 mg, 2.00 mmol), EDC (481 mg, 2.49 mmol), DMAP (101 mg, 0.833 mmol) and DIEA (870 μL, 4.99 mmol). The mixture was stirred for 26 h at room temperature. The solvent was removed under reduced pressure, and the residue was purified by flash chromatography on silica gel ($CHCl_3$: MeOH : AcOH = 20 : 1 : 0.2) to yield compound **9b** (493 mg, 1.25 mmol, 75 %).

^{1}H NMR (400 MHz, DMSO-d_6): δ 7.81 (d, 2H, J = 8.4 Hz), 7.43 (d, 2H, J = 8.4 Hz), 6.92 (m, 1H), 3.18 (m, 2H), 2.80–2.76 (m, 3H), 2.30 (t, 2H, J = 7.4 Hz), 2.09–2.05 (m, 2H), 1.56 (quintet, 2H, J = 7.2 Hz), 1.34 (s, 9H).

Compound **9c**

To a stirred solution of **9b** (252 mg, 0.638 mmol) in CH_2Cl_2 (5 mL) was added TFA (4 mL) at room temperature. The reaction solution was allowed to stir for 30 min. TFA was removed by twice co-evaporation with toluene (2 mL). To a solution of the residue in dry DMF (6 mL) was added **MTX-COOH**[11] (390 mg, 0.765 mmol), EDC (183 mg, 0.954 mmol), HOBt (146 mg, 0.956 mmol) and DIEA (1.11 mL, 6.37 mmol). The mixture was stirred for 22 h at room temperature. The solvent was removed under reduced pressure, and the residue was purified by flash chromatography on silica gel ($CHCl_3$: MeOH : AcOH = 15 : 1 : 0.8) to yield compound **9c** (397 mg, 0.505 mmol, 79 %).

^{1}H NMR (400 MHz, CD_3OD): δ 8.56 (s, 1H), 7.85 (d, 2H, J = 8.4 Hz), 7.75 (d, 2H, J = 9.2 Hz), 7.31 (d, 2H, J = 8.4 Hz), 6.85 (d, 2H, J = 9.2 Hz), 4.84 (s, 2H), 4.44–4.39 (m, 1H), 3.36 (t, 2H, J = 7.2 Hz), 3.24 (s, 3H), 2.81–2.76 (m, 2H), 2.33–1.98 (m, 9H), 1.67 (quintet, 2H, J =

7.2 Hz), 1.47 (s, 9H).

Compound **9d**

To a stirred solution of **9c** (50 mg, 61 µmol) in dry DMF (600 µL) was added iodoacetonitrile (5.27 µL, 72.6 µmol), DIEA (44.3 µL, 0.242 mmol). The mixture was stirred 24 h at room temperature. The solvent was removed under reduced pressure and the residue was purified by flash chromatography on silica gel ($CHCl_3$: MeOH = 15: 1) to yield compound **9d** (14.4 mg, 17.4 µmol, 29 %).

^{1}H NMR (400 MHz, CD_3OD): δ 8.56 (s, 1H), 7.88 (d, 2H, *J* = 8.8 Hz), 7.76 (d, 2H, *J* = 8.8 Hz), 7.45 (d, 2H, *J* = 8.8 Hz), 6.88 (d, 2H, *J* = 8.8 Hz), 4.85 (s, 2H), 4.79 (s, 2H), 4.43–4.40 (m, 1H), 3.26 (s, 3H), 2.86–2.81 (m, 4H), 2.31–2.13 (m, 6H), 2.05–1.96 (m, 1H) 1.72 (quintet, 2H, *J* = 7.0 Hz), 1.47 (s, 9H).

Compound **9**

To a stirred solution of **9d** (14.4 mg, 17.4 µmol) in DMF (200 µL) was added TFA (400 µL) at room temperature. The reaction solution was allowed to stir for 1 h. TFA was removed by twice co-evaporation with toluene (2 mL). To a solution of the residue in dry DMF (2 mL) was added $CuSO_4 \cdot 5H_2O$ (218 mg, 0.872 mmol), ascorbic acid (307 mg, 1.74 mmol) and Oregon Green 488 azide (6-isomer, 14.0 mg, 26.15 µmol) under Ar. The mixture was stirred for 1 h at room temperature. The solvent was removed under reduced pressure, and the residue was purified by RP-HPLC (gradient (A:B) 0 min; 20:80, 40 min; 60:40) to yield compound **9** (4.18 mg, 3.20 µmol, 18 %).

^{1}H NMR (400 MHz, DMSO-d_6): δ 10.8 (s, 2H), 9.27 (s, 1H), 9.07 (s, 1H), 8.70 (s, 1H), 8.64 (t, 1H, *J* = 5.6 Hz), 8.29 (d, 1H, *J* = 7.6 Hz), 8.15 (d, 1H, *J* = 8.0 Hz), 8.06 (d, 1H, *J* = 7.6 Hz), 7.97–7.95 (m, 1H), 7.89 (d, 2H, *J* = 8.8 Hz), 7.79 (s, 1H), 7.75 (d, 2H, *J* = 8.8 Hz), 7.65 (s, 1H), 7.48 (d, 2H, *J* = 8.4 Hz), 6.89 (d, 2H, *J* = 8.4 Hz), 6.82 (d, 2H, *J* = 9.2 Hz), 6.57 (d, 2H, *J* = 11.2 Hz), 4.90 (s, 2H), 4.87 (s, 2H), 4.32–4.28 (m, 1H), 4.24 (t, 2H, *J* = 7.0 Hz), 3.29 (t, 2H, *J* = 6.2 Hz), 3.24 (s, 3H), 3.19–3.14 (m, 2H), 2.80 (t, 2H, *J* = 7.0 Hz), 2.72–2.68 (m, 2H), 2.19–2.15 (m, 2H), 2.07–1.85 (m, 2H) 1.77–1.72 (m, 4H), 1.43–1.41 (m, 2H), 1.28–1.15 (m, 6H).

^{13}C NMR (150 MHz, DMSO-d_6): δ 173.7, 171.7, 171.5, 167.6, 166.7, 166.0, 164.4, 162.6, 155.7, 154.3, 153.0, 151.9, 151.8, 150.5, 148.6, 147.6, 147.5, 147.3, 147.2, 145.9, 145.8, 140.9, 135.5, 129.9, 129.5, 129.4, 128.9, 127.8, 127.6, 125.3, 125.2, 122.0, 121.6, 121.3, 116.3, 114.0 ($J_{C\text{-}F}$ = 21.6 Hz), 111.0, 107.8, 104.7, 82.2, 54.8, 52.1, 49.0, 39.9, 39.7, 39.6, 39.5, 39.3, 39.2, 39.0, 34.9, 34.5, 33.7, 31.9, 29.6, 28.7, 26.4, 25.8, 25.5, 23.9, 23.7, 1.1, 0.1.

HRMS (ESI) calculated for $C_{63}H_{61}F_2N_{15}O_{13}S$ ([M+H]$^+$) 1306.4335, found 1306.4314.

Synthesis of compound 10

Compound **10**

A solution of **10a**[5] (7.5 mg, 8.9 μmol) in TFA (1 mL) was stirred for 2 h at 0 °C. The solvent was removed by twice co-evaporation with toluene (2 mL). To a solution of the residue in dry DMF (0.5 mL) was added Oregon Green 488 Carboxylic acid, Succimidyl ester (OG-OSu) (5-isomer, 5.0 mg, 9.8 μmol) and DIEA (8.0 μL, 47 μmol). The mixture was stirred for 1 h at room temperature. The crude product was purified by RP-HPLC (CH_3CN : H_2O = 5 : 95 to 40 :60 for 50 min, without TFA) to yield **10** (2.1 mg, 1.96 μmol, 22 %) as yellow solid.

^{1}H NMR (400 MHz, DMSO-d_6): δ 8.88–8.86 (m, 1H), 8.56 (s, 1H), 8.49–8.47 (m, 1H), 8.21–8.15 (m, 2H), 8.10 (s, 1H), 7.90–7.85 (m, 1H), 7.71 (d, 2H, *J* = 8.8 Hz), 7.31 (s, 1H), 6.82 (d, 2H, *J* = 9.2 Hz), 6.64 (brs, 2H), 6.50–6.46 (m, 2H), 4.78 (s, 2H), 4.48 (t, 2H, *J* = 4.4 Hz), 4.28–4.20 (m, 1H), 3.78 (t, 2H, *J* = 4.4 Hz), 3.67–3.63 (m, 2H), 3.50–3.47 (m, 2H), 3.20 (s, 3H), 2.17–2.14 (m, 2H), 2.07–2.00 (m, 1H), 1.91–1.82 (m, 1H), 4H is overlapping with water peak.

^{13}C NMR (150 MHz, DMSO-d_6): δ 173.7, 171.5 (2C), 166.0 (2C), 162.7, 162.6, 155.1, 155.0, 150.8, 149.9, 149.1, 148.2 (2C), 146.0, 145.8, 141.2 (2C), 136.6 (2C), 135.8 (2C), 132.6, 129.3, 128.8 (2C), 128.1, 121.3 (2C), 121.2 (2C), 113.4 (2C), 111.0 (2C), 107.5 (2C), 104.6 (2C), 68.7, 67.5, 67.0, 66.7, 54.8, 54.6, 52.4, 37.9, 32.0, 27.8, 21.1.

HRMS (ESI) calculated for $C_{51}H_{46}F_2N_{12}O_{13}Na$ ([M+Na]$^+$) 1095.3152, found 1095.3168.

Synthesis of compound 11

Compound **11a**

To a suspension of **PU-NHBoc**[12] (57 mg, 0.10 mmol) in CH_2Cl_2 (1.6 mL) was added TFA (0.7 mL) at room temperature. The reaction solution was allowed to stir for 6 h. TFA was removed by twice co-evaporation with toluene (1 mL). To a stirred solution of this residue in dry DMF (1.2 mL) was added 4-sulfamoylbenzoic acid (24 mg, 0.12 mmol), EDC (23 mg, 0.12 mmol), HOBt (18 mg, 0.12 mmol) and DIEA (52 μL, 0.30 mmol). The mixture was stirred at room temperature for 12 h. The solvent was removed under reduced pressure.

The residue was dissolved with $CHCl_3$ (50 mL), washed with sat. $NaHCO_3$ aq. (5 mL) and brine (2 mL), dried over $MgSO_4$ and concentrated under reduced pressure. The residue was purified by flash chromatography on silica gel (MeOH:$CHCl_3$ = 1:10) to yield compound **11a** (23 mg, 35μmol, 35% over two steps) as a white powder.

^{1}H NMR (400 MHz, $CDCl_3$): δ 8.52 (m, 1H), 8.32 (s, 1H), 8.12 (d, 2H, *J* = 8.8 Hz), 8.05 (d, 2H, *J* = 8.8 Hz), 7.34 (s, 1H), 7.01 (s, 1H), 6.03 (s, 2H), 5.71 (brs, 2H), 4.93 (s, 2H), 4.36 (t, 2H, *J* = 6.4 Hz), 3.33 (q, 2H, *J* = 6.4 Hz), 2.08 (m, 2H).

Compound **11b**

A solution of the 4-(8-tert-butoxycarbonylamino-3,6-dioxaoctyl)aminocarbonyl butanoic acid[13] (25 mg, 69 μmol) in dry DMF (1.0 mL) was added **11a** (30 mg, 46 μmol), DMAP (8.4 mg, 69 μmol), DIEA (40 μL, 0.23 mmol) and EDC (13 mg, 69 μmol), and stirred at room temperature for 15 h. The solvent was removed under reduced pressure, and the residue was purified by flash chromatography on silica gel (MeOH:$CHCl_3$ = 1:15 to 1:10) to yield **11b** (35 mg, mixture as 6:1 = **11b**: 4-(8-tert-butoxycarbonylamino-3,6-dioxaoctyl) aminocarbonylbutanoic acid). No further purification.

Compound **11c**

To a solution of **11b** (15 mg, 15 μmol) in CH_2Cl_2 (1.0 mL) was added TFA (0.5 mL) at room temperature. The reaction solution was allowed to stir for 30 min. TFA was removed by twice co-evaporation with toluene (1.0 mL). To a stirred solution of this residue in dry DMF (1.0 mL) was added 5-carboxy-fluorescein diacetate *N*-succinimidyl ester (14 mg, 26 μmol) and DIEA (50 μL, 0.29 mmol). The mixture was stirred at room temperature for 1 h. The solvent was removed under reduced pressure, and the residue was purified by RP-HPLC (gradient (A:B) 0 min; 45:55, 40 min; 65:35) to yield **11c** (10 mg, 7.4 μmol, 50%) as a white solid.

^{1}H NMR (400 MHz, CD_3OD): δ 8.45 (s, 1H), 8.20 (dd, 2H, *J* = 8.0, 1.6 Hz), 8.06 (d, 2H, *J* = 8.8 Hz), 7.95 (d, 2H, *J* = 8.8 Hz), 7.40 (s, 1H), 7.34 (d, 1H, *J* = 8.0 Hz), 7.18–7.16 (m, 2H), 7.15 (s, 5H), 6.91–6.85 (m, 4H), 6.05 (s, 2H), 4.38 (t, 2H, *J* = 7.2 Hz), 3.71–3.58 (m, 15H), 3.53–3.44 (m, 5H), 2.30 (s, 6H), 2.28–2.20 (m, 4H), 2.12 (t, 2H, *J* = 7.2 Hz), 1.74 (quintet, 2H, *J* = 7.2 Hz).

HRMS (ESI) calcd for $[C_{58}H_{55}I_1N_9O_{17}S_2]^+$ $(M+H)^+$: *m/z* 1340.2197, found 1340.2171.

Compound **11**

To a solution of compound **11c** (10 mg, 7.4 μmol) in dry DMF (0.8 mL) was added

DIEA (10 µL, 60 µmol) and iodoacetonitrile (2 µL, 30 µmol). The mixture was stirred at room temperature for 24 h in the dark. The solvent was removed under reduced pressure, and the residue was purified by RP-HPLC (gradient (A:B) 0 min; 50:50, 40 min; 70:30) to yield compound **9** (4.8 mg, 3.4 µmol, 48%) as a white solid.

^{1}H NMR (400 MHz, CD_3CN): δ 8.37 (s, 1H), 8.25 (s, 1H), 8.17 (dd, 1H, *J* = 8.0, 1.6 Hz), 8.05 (d, 2H, *J* = 8.8 Hz), 7.99 (d, 2H, *J* = 8.8 Hz), 7.59 (m, 1H), 7.54 (m, 1H), 7.35–7.32 (m, 2H), 7.13 (d, 2H, *J* = 2.0 Hz), 6.98 (s, 1H), 6.91–6.84 (m, 4H), 6.51 (m, 1H), 6.01 (s, 2H), 4.74 (s, 2H), 4.30 (t, 2H, *J* = 7.2 Hz), 3.64–3.51 (m, 10H), 3.45 (t, 2H, *J* = 5.6 Hz), 3.35 (q, 2H, *J* = 6.4 Hz), 3.22 (q, 2H, *J* = 5.6 Hz), 2.69 (t, 2H, *J* = 7.2 Hz), 2.26 (s, 6H), 2.08 (m, 2H), 1.78–1.71 (m, 2H).

^{13}C NMR (100 MHz, CD_3CN): δ 172.9, 170.1 (2C), 169.1, 166.5 (2C), 166.3, 155.8, 153.6 (2C), 152.4 (2C), 151.7, 151.3, 151.1, 150.7, 150.4, 145.1, 141.3, 141.0, 138.0, 135.7 (2C), 130.1 (2C), 129.3 (2C), 129.1 (2C), 127.4, 127.0, 125.2, 124.7, 120.4, 120.1, 119.4 (2C), 117.0, 116.7, 114.1, 111.5 (2C), 104.0, 93.4, 82.5, 70.9 (2C), 70.3, 70.0, 43.0, 40.8, 39.9, 37.7, 35.9, 35.1, 34.8, 29.5, 21.2 (2C), 21.1.

HRMS (ESI) calcd for $[C_{60}H_{55}I_1N_{10}O_{17}S_2Na]^+$ $(M+Na)^+$: *m/z* 1401.2125, found 1401.2118.

Synthesis of compound 12

Compound **12a**

To a solution of benzenesulfonamide (518 mg, 3.30 mmol), monomethylglutarate (438 mg, 3.00 mmol), DMAP (439 mg, 3.60 mmol) and DIEA (1.88 mL, 10.8 mmol) in dry DMF (15 mL) was added EDC (690 mg, 3.60 mmol) at room temperature. The reaction solution was allowed to stir for 24 h. The solvent was removed under reduced pressure. The residue was dissolved with EtOAc (100 mL), washed with 1N HCl (15 mL), water (15 mL) and brine (5 mL), dried over $MgSO_4$ and concentrated under reduced pressure. The residue was purified by flash chromatography on silica gel ($MeOH:CHCl_3$ = 1:20) to yield compound **12a** (573 mg, 2.01 mmol, 67%) as a white powder.

^{1}H NMR (400 MHz, CD_3OD): δ 8.00–7.98 (m, 2H), 7.67 (m, 1H), 7.59–7.55 (m, 2H), 3.60 (s, 3H), 2.27 (t, 2H, *J* = 7.2 Hz), 2.24 (t, 2H, *J* = 7.2 Hz), 1.77 (quintet, 2H, *J* = 7.2 Hz).

Compound **12b**

To a solution of **12a** (200 mg, 0.70 mmol) in MeOH (2.8 mL) and H_2O (0.7 mL) was added LiOH (84 mg, 3.5 mmol) for room temperature. The reaction solution was allowed

to stir for 2 h. The reaction was quenched by the addition of EtOAc (60 mL) and 1N HCl (5 mL). The organic layer was washed water (5 mL) and brine (5 mL), dried over $MgSO_4$, and concentrated under reduced pressure to yield compound **12b** (149 mg, 0.55 mmol, 78%) as a white solid.

^{1}H NMR (400 MHz, CD_3OD): δ 8.01–7.98 (m, 2H), 7.67 (m, 1H), 7.59–7.55 (m, 2H), 2.28 (t, 2H, *J* = 7.2 Hz), 2.21 (t, 2H, *J* = 7.2 Hz), 1.75 (quintet, 2H, *J* = 7.2 Hz).

Compound **12c**

To a suspension of **PU-NHBoc** (12 mg, 0.021 mmol) in CH_2Cl_2 (1 mL) was added TFA (0.5 mL) at room temperature. The reaction solution was allowed to stir for 30 min. TFA was removed by twice co-evaporation with toluene (1 mL). To a stirred solution of this residue in dry DMF (1 mL) was added **12b** (6.8 mg, 0.025 mmol), EDC (4.8 mg, 0.025 mmol), DMAP (3.0 mg, 0.025 mmol) and DIEA (22 µL, 0.12 mmol). The mixture was stirred at room temperature for 24 h. The solvent was removed under reduced pressure. The residue was purified by flash chromatography on silica gel (MeOH:$CHCl_3$ = 1:15 to 1:10) to yield compound **12c** (15 mg, 0.020 mmol, 98% over two steps) as a white solid.

^{1}H NMR (400 MHz, $CDCl_3$:CD_3OD = 1:1): δ 8.20 (s, 1H), 8.01 (d, 2H, *J* = 8.0 Hz), 7.66–7.53 (m, 3H), 7.41 (s, 1H), 7.09 (s, 1H), 6.08 (s, 2H), 4.26 (t, 2H, *J* = 7.2 Hz), 3.23 (t, 2H, *J* = 7.2 Hz), 2.29 (t, 2H, *J* = 7.2 Hz), 2.23 (t, 2H, *J* = 7.2 Hz), 2.03 (quintet, 2H, *J* = 7.2 Hz), 1.87 (quintet, 2H, *J* = 7.2 Hz).

Compound **12**

To a solution of compound **12c** (15 mg, 20 µmol) in dry DMF (0.5 mL) was added DIEA (28.0 µL, 164 µmol) and iodoacetonitrile (6.0 µL, 82 µmol). The mixture was stirred at room temperature for 24 h in the dark. The solvent was removed under reduced pressure, and the residue was purified by RP-HPLC (gradient (A:B) 0 min; 45:55, 50 min; 70:30) to yield **12** (9.9 mg, 12 µmol, 64%) as a brown solid.

^{1}H NMR (400 MHz, CD_3CN): δ 8.26 (s, 1H), 7.97 (d, 2H, *J* = 8.4 Hz), 7.76 (t, 1H, *J* = 7.6 Hz), 7.64 (t, 2H, *J* = 8.4 Hz), 7.43 (s, 1H), 7.06 (s, 1H), 6.48 (brs, 1H), 6.04 (s, 2H), 4.70 (s, 2H), 4.23 (t, 2H, *J* = 7.2 Hz), 3.11 (q, 2H, *J* = 6.4 Hz), 2.72 (t, 2H, *J* = 6.4 Hz), 2.10 (t, 2H, *J* = 7.2 Hz), 1.84–1.74 (m, 4H).

^{13}C NMR (100 MHz, CD_3CN): δ 173.2, 173.0, 151.7, 151.3, 151.2, 150.8, 150.5, 145.0, 139.2, 135.8, 130.7 (2C), 128.7 (2C), 127.0, 120.4, 120.1, 116.7, 114.3, 104.0, 93.7, 43.0, 36.9, 36.0, 35.3, 34.7, 30.0, 21.2.

HRMS (ESI) calcd for $[C_{28}H_{28}I_1N_8O_6S_2]^+$ $(M+H)^+$: *m*/*z* 763.0612, found 763.0598.

Supplementary References

1. Taldone, T. *et al.* Experimental and structural testing module to analyze paralogue-specificity and affinity in the Hsp90 inhibitors series. *J. Med. Chem.* **56,** 6803–6818 (2013).
2. Patel, P. D. *et al.* Paralog-selective Hsp90 inhibitors define tumor-specific regulation of HER2. *Nat. Chem. Biol.,* **9**, 677–684 (2013).
3. Kitz, R. & Wilson, I. B. Esters of methanesulfonic acid as irreversible inhibitors of acetylcholinesterase. *J. Biol. Chem.*, **237**, 3245–3249 (1962).
4. Tamura, T., Tsukiji, S. & Hamachi, I. Native FKBP12 engineering by ligand-directed tosyl chemistry: labelling properties and application to photo-cross-linking of protein complexes in vitro and in living cells. *J. Am. Chem. Soc.* **134**, 2216–2226 (2012).
5. Fujishima, S., Yasui, R., Miki, T., Ojida, A. & Hamachi, I. Ligand-directed acyl imidazole chemistry for labelling of membrane-bound proteins on live cells. *J. Am. Chem. Soc.* **134**, 3961–3964 (2012).
6. Takaoka, Y., Nishikawa, Y., Hashimoto, Y., Sasaki, K. & Hamachi, I. Ligand-directed dibromophenyl benzoate chemistry for rapid and selective acylation of intracellular natural proteins. *Chem. Sci.,* **6,** 3217–3224 (2015).
7. Tanaka, T., Kamiya, N. & Nagamune, T. N-terminal glycine-specific protein conjugation catalyzed by microbial transglutaminase. *FEBS Lett.*, **579**, 2092–2096 (2005).
8. Young, J. C., Schneider, C. & Hartl, F. U. In vitro evidence that hsp90 contains two independent chaperone sites. *FEBS Lett.* **418,** 139–143 (1997).
9. Keenan, T., Yaeger, D.R., Courage, N.L., Rollins, C.T., Pavone, M.E., Rivera,V.M.,Yang, W., Guo, T., Amara, J.F., Clackson, T., Gilman, M. & Holt, D.A. Synthesis and activity of bivalent FKBP12 ligands for the regulated dimerization of proteins. *Bioorg. Med. Chem.*, **6**, 1309–1335 (1998).
10. Ando, T., Tsukiji, S., Tanaka, T., Nagamune, T. Construction of a small-molecule-integrated semisynthetic split intein for *in vivo* protein ligation. *Chem. Commun.*, **47**, 4995–4997 (2007).
11. Nagy, A., Szoke, B., Schally, A. V., Selective coupling of methotrexate to peptide hormone carriers through a gamma-carboxamide linkage of its glutamic acid moiety: benzotriazol-1-yloxytris(dimethylamino)phosphonium hexafluorophosphate activation in salt coupling. *Proc. Natl. Acad. Sci. USA*, **90**, 6373–6376 (1993).
12. Yoshii, T., Mizusawa, K., Takaoka, Y., Hamachi, I. Intracellular protein-responsive supramolecules: protein sensing and in-cell construction of inhibitor assay system. *J. Am.*

Chem. Soc., **136**, 16635–16642 (2014)

13. Keller, M. *et al.*, Red-fluorescent argininamide-type NPY Y1 receptor antagonists as pharmacological tools. *Bioorg. Med. Chem.* **19**, 2859–2878 (2011).
