# Supplementary material for: Rapid labelling and covalent inhibition of intracellular native proteins using ligand-directed N-acyl-N-alkyl sulfonamide
Source: Nat Commun. 2018 May 14;9:1870. doi: 10.1038/s41467-018-04343-0 (PMC5951806; doi:10.1038/s41467-018-04343-0)
Supplement: Supplementary file 3 — Description of Additional Supplementary Files [file 41467_2018_4343_MOESM3_ESM.pdf]

Description of Additional Supplementary Files

File Name: Supplementary Data 1
Description: List of proteins identified in the LC-MSMS analysis of proteins labelled with 11.
